# SELAMAT: A New Secure and Lightweight Multi-Factor Authentication Scheme for Cross-Platform Industrial IoT Systems

**DOI:** 10.3390/s21041428

**Published:** 2021-02-18

**Authors:** Haqi Khalid, Shaiful Jahari Hashim, Sharifah Mumtazah Syed Ahmad, Fazirulhisyam Hashim, Muhammad Akmal Chaudhary

**Affiliations:** 1 Department of Computer and Communication Systems Engineering, Faculty of Engineering, Universiti Putra Malaysia, Serdang 43400, Malaysia; s_mumtazah@upm.edu.my (S.M.S.A.); fazirul@upm.edu.my (F.H.); 2 Department of Electrical and Computer Engineering, College of Engineering and Information Technology, Ajman University, Ajman 346, United Arab Emirates; m.akmal@ajman.ac.ae

**Keywords:** multi-factor authentication, fog computing, industrial IoT, fog node, cross-platform

## Abstract

The development of the industrial Internet of Things (IIoT) promotes the integration of the cross-platform systems in fog computing, which enable users to obtain access to multiple application located in different geographical locations. Fog users at the network’s edge communicate with many fog servers in different fogs and newly joined servers that they had never contacted before. This communication complexity brings enormous security challenges and potential vulnerability to malicious threats. The attacker may replace the edge device with a fake one and authenticate it as a legitimate device. Therefore, to prevent unauthorized users from accessing fog servers, we propose a new secure and lightweight multi-factor authentication scheme for cross-platform IoT systems (SELAMAT). The proposed scheme extends the Kerberos workflow and utilizes the AES-ECC algorithm for efficient encryption keys management and secure communication between the edge nodes and fog node servers to establish secure mutual authentication. The scheme was tested for its security analysis using the formal security verification under the widely accepted AVISPA tool. We proved our scheme using Burrows Abdi Needham’s logic (BAN logic) to prove secure mutual authentication. The results show that the SELAMAT scheme provides better security, functionality, communication, and computation cost than the existing schemes.

## 1. Introduction

**Figure 1:**
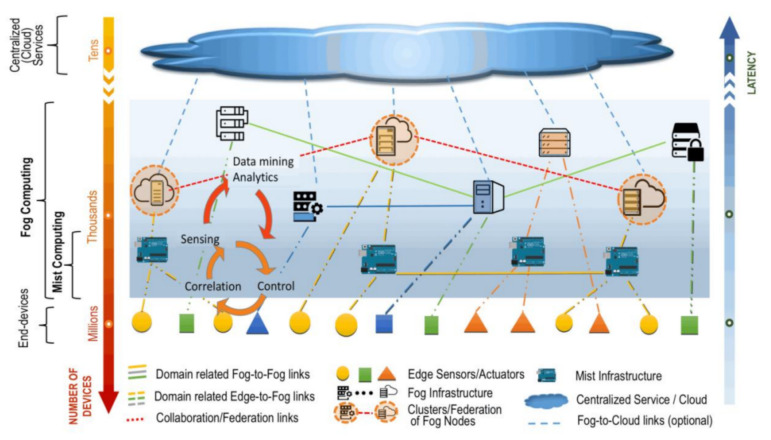
Fog computing supporting a cloud base for smart end-devices [9].

The Internet of things (IoT) has gained tremendous popularity in the last decade with the advent of many powerful, low-cost devices such as sensors, RFIDs, etc., coupled with various communication media. Recently, the implementation of IoT in industries with Cyber-Physical System (CPS) as a part of the world of production and network connectivity is known as Industrial IoT (IIoT) [1]. The integration combines industrial devices equipped with communication, sensors, and Internet-connected actuator modules [1]. The devices are responsible for sensory data capture, environmental and industrial conditions tracking, and raw goods transport. However, it is estimated that the industrial IoT market will hit $123.89 billion by 2021 [2]. Industrial IoT (IIoT) can significantly enhance communication, efficiency, scalability, time savings, and cost savings for industrial sectors. Interoperability between devices and machines using different protocols with different architectures and the security of such protocols and data generated with these devices is the primary concern for IIoT [2,3,4]. As has been stated, industrial devices capture, store, transmit, or exchange large amounts of highly sensitive consumer information. The attacker can intercept and alter this transmitted data. These attacks threaten confidentiality in the information collected and transmitted, leading to less trust in the entire system [5]. Therefore, it is essential to implement essential security features, such as confidentiality and integrity. Constrained devices, however, are the primary security considerations for IoT and IIoT applications. These devices are typically limited in computing power, storage capacity, and energy consumption. Therefore, it is a challenge to use some high computational cryptographic algorithms, which usually require more computation costs [1,6,7]. The National Institute of Standards and Techniques (NIST) reports that the fog computing architecture consists of three layers of edge devices, fog nodes, and a cloud layer. The edge device is the initial layer of fog architecture [8]. It is used to collect data and environmental monitoring by various industrially smart IoT devices (sensors and actuators). The fog nodes are context-conscious and support a shared data and communication system. The last layer consists of the cloud server, which stores data for potential use [9]. Services can be hosted in fog computing at end devices such as access points, as shown in Figure 1.

Fog providers may be different parties because of various deployment solutions; the existing infrastructure for fog can be used for wireless carriers (e.g., GSM) that control home or cellular base stations. Fog infrastructure can also be developed by cloud service providers needing their cloud services to the network edge. Because of the lack of authentication services, a rogue fog node/service will be a fog device or fog instance, which claims to be legitimate and attempts to control edge’s fog nodes [10]. For example, a fog administrator may permit an insider attack not to instantiate a legitimate attack but to instantiate a rogue fog instance. For example, in an insider attack, a fog administrator can handle fog instances but can instead instantiate a rogue fog instance [9]. After communication, an adversary can manage incoming or outgoing user or cloud requests, capture or control user data stealthily, and initiate further attacks quickly. A fake fog node or server is a serious threat to the security and privacy of user data. Likewise, in 2012, Fire-base Google was launched to allow a front-end application to connect directly to a back-end database. However, they discovered that the Fire-base is vulnerable to the Stuxnet attack due to the absence of authentication and authorization [11]. Thus, due to an exposed interconnection between the edge devices and the fog node, authenticating users/devices and ensuring platform security becomes a huge challenge. The devices often have a low computational capacity and low power consumption, particularly in the IoT system, which requires reducing authentication and encryption costs while ensuring information security [12]. Therefore, cross-platform authentication in cloud computing has not been considered before, and this gives rise to the problem of trustiness between cross-platforms in fog computing. Cross-platforms are places where multiple fog nodes authenticate mutually and exchange data [13,14,15,16,17]. Hence, developing lightweight cryptographic protocols to protect Industrial IoT devices against vulnerable attacks and satisfying device constraints are needed.

Thus, we propose a secure and lightweight multi-factor authentication scheme for cross-platform Industrial IoT systems (SELAMAT). The scheme intends to improve the security and establish secure communication between the edge devices and fog nodes. The SELAMAT scheme uses the AES-ECC algorithm to design an efficient key management system. AES (Symmetric Key Encryption Scheme) for the ECC Message Encryption (A-Symmetric Key Encryption Scheme) for the Secure Key Management mechanism is used in combination with data hiding to provide strong encryption and decryption requirements by using the advantages of both the cryptographic schemes. With our multi-factor authentication (MFA), three types of factors are used: Username/Password (something you know), smart card (something you have), and biometric (Fingerprint). Our MFA secures the user information from password guessing attacks, session attacks, and impersonation attacks. It provides layered security, making access in the fog node more difficult for unauthorized users to a target such as the physical location, device, network, application, or database.

## 2. Industrial IoT Security Requirements and Issues

The industrial Internet of things improves the efficiency, scalability, and security of the industrial environment applications. In such sensitive applications, introducing the resource-constrained IoT devices might bring essential security and privacy concerns.

### 2.1. Security Requirements

Several types of research [18,19] have underlined security requirements that must be considered in IIoT, particularly fog nodes and sensors. We further identify the most critical security and privacy requirements:

- Availability: The network infrastructure, devices (e.g., sensors), and fog nodes that handle the control and optimization queries should be continually available. Besides, unauthorized users should not deny allowing authorized users to handle queries.
- Confidentiality: The data and queries between edge nodes and fog nodes exchanged are confidential and must not be revealed by unauthorized third parties.
- Integrity: The type of data sharing between edge devices and fog nodes improves energy transmission decision making. For better decision-making, the integrity of these data is fundamental. We also need to deal with injection attacks that aim to inject false measures into the fog computing infrastructure that could interrupt decisions.
- Authenticity: Authentication of ubiquitous IoT connectivity is based on the nature of Industrial IoT environments in which communication between equipment and equipment Machine-to-Machine (M2M), between man and device, or between user and other would be possible. The authorizations property allows only authorized entities (any authenticated entity) to carry out certain network operations. It is necessary to design a secure authentication scheme to prevent unauthorized users from accessing the nodes.
- Non-repudiation: Any party in the system between the utilities’ fog node servers and the edge nodes must not deny that they subsequently have not received such data or control commands.
- Privacy: Fog computing infrastructure information includes fine-grained data about users and even industrial machines. These data reveal information about the activities of customers in industries and companies. It is compulsory to encrypt and make these data untraceable.

### 2.2. Security Issues

Fog computing should withstand some security challenges in the Industrial IoT setting. We present the relevant ones as follows:

- Limitations of information system technology: The number of attacks is increasing because many industries are interconnected with cloud computing, which may affect the fog computing network’s availability. The integrity of data, confidentiality, and privacy; spoofing servers; injection; DoS/DDoS attacks; impersonation attacks; and replay attacks, among others, are just some examples of attacks.
- Data sensitivity and privacy: The information shared between the network node and other fog nodes involves sensitive customer-specific information such as object tracking, power consumption, real-time data streaming, and performance monitoring. Neighbors should not leak this information while keeping it exploitable across fog nodes.
- Lack of Authentication: Fog computing nodes must be securely designed. They must verify information from a known source and ensure that it was not corrupted to avoid introducing some threats. The weak authentication mechanism for industrial sectors might allow attackers to impersonate a legitimate user. For example, an adversary may execute a password guessing attack, man-in-the-middle, or replay attack to access the targeted node. Therefore, a secure authentication scheme must be designed to prevent such attacks.
- Lack of data transmission encryption: The data exchange between edge devices and fog nodes is typical, not encrypted, and transmitted through a public channel. In this case, the attacker can still capture the network’s data by using a simple network sniffer to monitor the connection between the user and the IIoT device. The attacker also can record the message and obtain the edge device information to perform a replay attack. The non-encrypted form allows the attacker to gather information about the targeted node, such as its database used by the node/device.
- Complexity: Researchers have proposed several authentication schemes for the IIoT environment, but those schemes are mostly based on cryptographic techniques requiring a high computational cost. Some of the cryptographic techniques use an extensive operation, such as the identity-based verification and multiplication operation. Since industrial IoT devices are limited resources with low power, a lightweight authentication scheme must be designed for IIoT suitability.

## 3. Related Works

Many researchers recently focused on providing secure authentication for industrial IoT systems. However, Chen et al. (2020) proposed a fog node Authentication Secure Authenticated Key Exchange Scheme [20]. Moreover, the proposed scheme uses only such basic operations because of limited resources of fog nodes and users, such as multiplication of elliptic curve cryptography point operations, bit-wise exclusive OR, and hash-only functions, instead of other complex algorithms. They argued that their system overcomes the risk of a temporary secret leakage attack. They proposed a three-step authentication system of: (1) the user registration phase, (2) fog server registration phase, and (3) login and authentication phase. However, due to a large amount of computation and communication, it involves heavy calculations due to public cryptography and signature algorithms or other time-consuming calculations (e.g., bilinear pairings). Munir et al. (2018) [21] proposed a biometric smart card authentication scheme based on pin and fingerprint identification in fog computing. In Phase 1, the user enters his information in two phases. Simultaneously, the pin is encrypted with DES and the fingerprint, which uses a robust mathematical algorithm that is not invertible and where both are stored on the fog server and smart card. In Phase 2, the user inserts the card and receives the verified information. The unauthorized individual has access to these data if some government or other source leaks the biometric data. However, privacy issues have been increased because an individual’s unique identity is a biometric blueprint. Since the template cannot be decoded back to biometric data, it can be used to track individuals if a database links the user to a specific biometric prototype, so the user’s operation can be tracked unlawfully. Such threats must be tackled, and cancellable biometrics are a potential solution.

Rahman et al. (2019) [22] suggested that an enhanced mutual authentication security scheme be addressed based on an advanced encryption protocol and hashed message authentication code for fog computing. The authors tried to avoid the mid-attack in interactions between the fog user and registration authority. The attacker compromises the user’s identity by sending the identity to the registering authority as fog users obtain from the registering authority the master secret key. However, this work still built session keys with a long-term master secret. A different scheme of mutual authentication is proposed for fog-based computer environments with constrained devices [23]. The proposed scheme, called Octopus, needs a long-lived secret key to authenticate with any fog server. However, it is about setting up and resuming the session. Session hijacking on the transport layer will result in a DoS attack. An attacking node may individually identify the victim node to continue between the two nodes. The nodes that are communicating may need to re-transmission messages by altering sequence numbers [22,24,25]. In [26], the authors provided secure key management and user authentication scheme called the SAKA-FC. It is defined as a secure communication protocol that supports fog and uses a one-way hash function and XOR that is bitwise supported by IoT-resource-driven devices. The scheme suffers from controlling the privileged insider attack and cannot provide a secure environment to compromise the attack. It should be noted that the authentication of users and binding agreements are not secured against future attacks. The scheme is not as lightweight as it requires more complex computing and communication. As a result, the scheme proposed for this environment will not correctly authenticate users in cloud-driven IIoT environments [27].

Similarly, Wang et al. [12] designed an anonymous lightweight authentication protocol for multi-level architecture fog-based applications. The protocol dynamically updates both sides of the session keys and ensures anonymous user authentications. The scheme proposed a key group protocol for management. The server can share with a specific attribute the key to desired communication nodes, and a private key between the two fog nodes can be created and updated without having to leak keys on the servers. However, the available group key management systems are not enough for mobile devices and require time-consuming computations. Moreover, He et al. [28] presented a new Mobile Healthcare Social Networks (MHSNs) handshake scheme. The scheme is based on hierarchical identity-based cryptography. The system consists of three tiers, while the highest level is a trusted authority using the Schnorr signature scheme to generate private keys to participating health centers. The second level is registered health centers with the Schnorr signature system, responsible for generating private keys. The third level is machine patients performing symptoms matching the cross-domain handshake. The system’s goal was to allow two users registered in separate healthcare centers to conduct a symptom-matching cross-domain handshake. However, the scheme requires more computational effort because of its identity-based cryptographic technologies and lack of reliability due to resource-constrained devices (such as the elliptic curve) being more time consuming as they come with some operations [29,30].

In 2019, Jia et al. [31] proposed an AKE Scheme for an IoT-based fog computing health care system. Compared to conventional medical systems, data from consumer devices and sensors are transmitted via fog nodes in the fog layer instead of via the cloud. Fog nodes process, transfer, and store data to the end-user and return results. However, we found that this AKE device is vulnerable to a temporary secret attack [20]. M. Akram et al. [32] enhanced the security features by proposing an anonymous three-factor authentication scheme for multi-servers. The scheme designed was based on the elliptic curve cryptography, and the biometric information is verified by the user and the server separately. The registration center in their scheme is involved in the authentication phase and has separate responsibilities with the server. Likewise, H. Tan et al. [33] designed a pairing-free homographic authentication and key management scheme for VANET dynamic cross-platform authentication. The scheme used certificateless cryptography for mutual authentication and homomorphic key management. On each active validation, dynamic updating to anonymous vehicle identity is performed to achieve privacy preservation. It mainly focuses on solving the heavy bandwidth consumption and high latency. However, their scheme is vulnerable to specific passive and active attacks such as server spoofing attacks and DoS attacks.

Venčkauskas et al. (2019) [34] presented the secure Self-Authenticable Data Transfer Protocol to address the issue of secure communication between resource-constrained devices. The authors proposed a new lightweight, secure, and authenticable transfer protocol for communication between the edge nodes and the fog nodes. Instead of UDP (User Datagram Protocol) and DTLS (Datagram Transport Layer Security) protocols, the primary purpose of the proposed protocol is to use CoAP (Constrained Application Protocol) as a secure transport. SSATP only uses primitive symmetric cryptography, allowing small devices with memory and low-end processing capacities to be easily implementable. However, DTLS must be excluded from the critical quality to suit the smart cities’ resource-intensive sensor nodes [12,35,36]. From the discussion above, we can see that most of the authentication schemes are still not satisfying the fog computing requirements. Thus, a lightweight and secure authentication scheme for the fog computing architecture is urgently needed. The comparison of the related authentication scheme is illustrated in Table 1.

## 4. Preliminaries

This section introduces important cryptographic principles and basic knowledge. The elliptic cryptosystem (ECC) and AES-ECC encryption and decryption are specified. Related notes, system models, security requirements, and network assumptions are then specified.

### 4.1. The Elliptic Curve Cryptography

Let p>3 be the large prime and $F_{p}$ be the finite field with order *p*, where $a,b\in F_{p}$ satisfy the following equation: $4a^{3}+27b^{2}\left(modp\right)\neq 0$. The elliptic curve $E_{p}\left(a,b\right)$ over the finite field $F_{p}$ is defined with the following equation:

(1) $$y^{2}=x^{3}+ax+b\;\mathrm{mod}\;p,$$

where $\left(x,y\right)\in F_{p}$. The additional operation on the curve is known as the doubling point of the two points. The addition of point is otherwise specified. All points on the curve and the point at infinity are a group of abelites $E(F_{p})$ additives. Please note that ∞=(−∞) performs as the identity element.

**Definition** **1** **(Computational Diffie–Hellman Problem (CDHP))**. Given $P,aP,bP\in G_{1}$ for $a,b\in Z_{q}^{*}$ where P is a generator of $G_{1}$, the advantage in computing ab P to solve the CDHP problem for any probabilistic polynomial-time (PPT) algorithm A is negligible, which can be defined as:

(2) $$Adv_{A,G_{1}}^{CDHP}=Pr\left[A\left(P,aP,bP\right)\to :a,b\in Z_{q}^{*}\right]$$

**Definition** **2** **(Elliptic Curve Discrete Logarithm Problem (ECDLP))**. Given $P,Q\in G_{1}$, where Q=aP. The advantage in finding the integer $a\in Z_{q}^{*}$ to solve the ECDLP problem for any probabilistic polynomial-time (PPT) algorithm A is negligible, which can be defined as:

(3) $$Adv_{A,G_{1}}^{CDHP}Pr\left[\left(P,aP\right)\to a:a\in Z_{q}^{*}\right].$$

### 4.2. AES-ECC Encryption/Decryption

**Figure 2:**
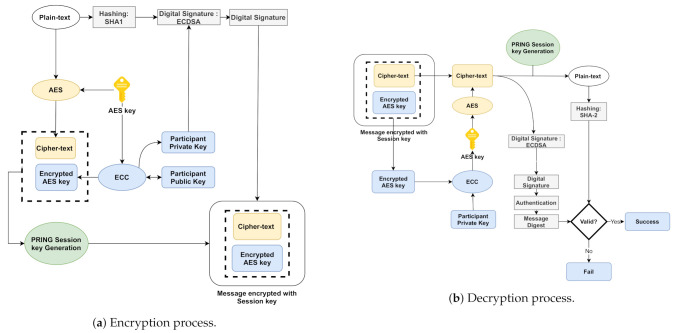
AES-ECC Encryption/Decryption.

In this section, we explain the process of the AES-ECC algorithm for efficient key generation and secure user data transmission. The ECC is utilized to encrypt and transfer the Private Keys as AES Private Keys while AES encrypts the plain text (communication data). The process is applied when an entity needs to encrypt/decrypt the message [37,38]. The encryption/decryption processes are shown in Figure 2, and the steps are explained as follow:

1. Data are the users’ information, i.e., their identities, passwords, and biometrics.
2. SHA-2 is used to produce a data summary.
3. ECC-related sender private key and ECDSA module are used to produce a digital signature.
4. According to the AES encryption module (AES private key), digital signature encryption and data to be submitted are encrypted. Then, the ciphertext data and ciphertext signature are encrypted.
5. The AES private key is encrypted by the ECC encryption module, and then the key-ciphertext is generated.
6. All the ciphertexts are packed and sent via the cross-d system network to the receiver.
7. Therefore, the sender uploads the ciphertext to the authentication server.
8. When the receiver receives the ciphertext, the receiver uses its private key to decrypt the AES key, and then decrypts the AES key data-ciphertext and signature-ciphertext. He/she uses the public key to check the signature, digest the message, and then get the plaintext by using the SHA-2 algorithm. If the message digest and plaintext are the same, the data are valid and available; otherwise, they are invalid.

## 5. SELAMAT Scheme

**Figure 3:**
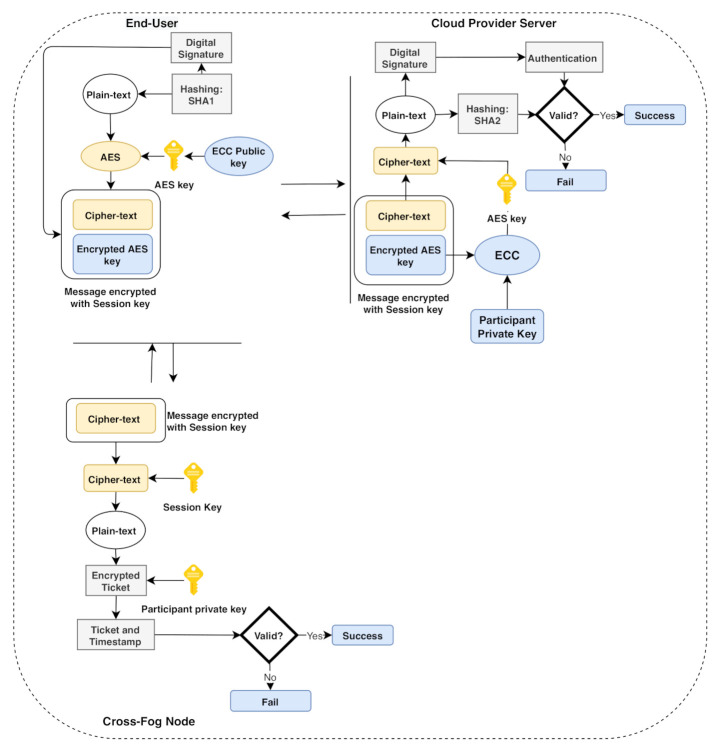
The system architecture for SELAMAT.

**Figure 4:**
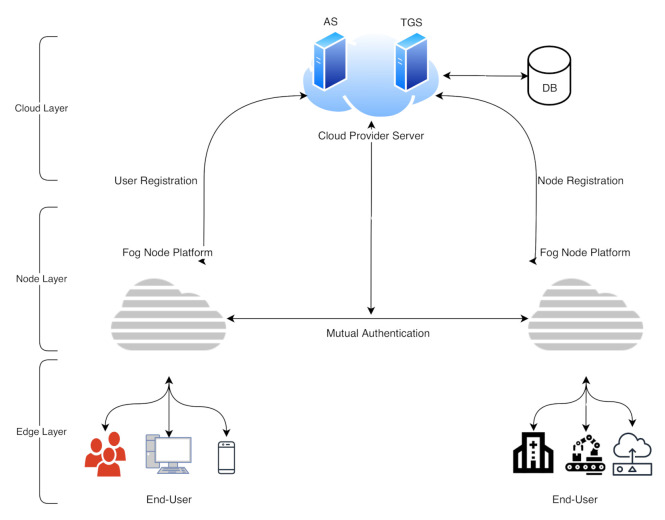
The mutual authentication scheme in fog computing architecture.

This section proposes a multi-factor authentication scheme for industrial IoT (IIoT) to establish secure communication between the edge devices and the fog node. The system backend architecture is shown in Figure 3; our scheme is based on a smart card, username/password, and a biometric (fingerprint). The scheme adopts the combination of the AES-ECC algorithm for secure key management. It provides a secure mutual authentication among the edge and the fog server; the mutual authentication diagram is illustrated in Figure 4. The proposed scheme comprises five phases, i.e., the setup phase, user registration phase, fog node registration phase, login phase, and the authentication phase. We explain the scheme phases as follows.

### 5.1. Setup Phase

The Cloud Provider Server (CPS) selects a κ-bit prime number p and an elliptic curve $E/F_{p}$. The generated elliptic curve group *G* has a generator *P*. Then, CPS selects a random integer $S\in Z_{q}^{*}$ as the system master key and calculates the system public key accordingly PK=S.p. After that, CPS choose asymmetric encryption/decryption pairs $E\left\{.\right\}/D\left\{.\right\}$, and cryptographic collision-resistant hash function H(.). Note that the AES shared private key is used to encrypt and decrypt the user information while being transmitted among the entities. CPS later publishes the system public parameters $\left\{G,p,q,PK,H(.)\right\}$ and keep the system master key S secretly. Moreover, when the user enters his/her information, it is then encrypted using the AES symmetric algorithm. The AES key is a shared private key between the sender and the receiver, which is used to encrypt the user information, and then it is encrypted with the ECC public key shared earlier with the user. The cipher-text is then transmitted to the AS in the CPS for verification. The used notions in the proposed scheme are shown in Table 2.

### 5.2. User Registration Phase

**Figure 5:**
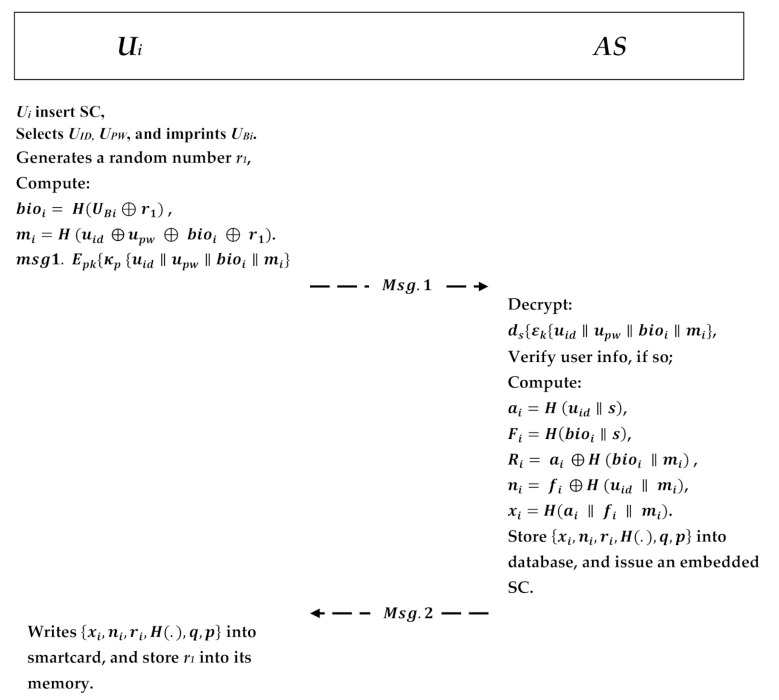
User registration phase.

In this phase, the user must register himself/herself at the cloud provider server to access the data that he wants to use. After that, the user Ui is issued a smart card; the authentication server (AS) in the CPS stores the user has protected biometric information Ui in its database. The registration is transmitted securely to obtain the smart card SC. The user is not required to send his/her information in plaintext because the proposed scheme utilizes asymmetric encryption/decryption pair in which the information will be encrypted. In addition, that transmitted information is sensitive and will be handed to the server in a masked manner using the hash function for other security matters to prevent an insider attack. The user will firstly select his/her unique identity and password and input his/her biometric information. Upon receiving the request, the authentication server will decrypt the message and verify the given information whether he/she already exists in the server database or not. If $U_{i}$ already exists at the server, it will inform the user that the identity exists and choose another. Otherwise, the AS will start user registration by performing the following steps, as shown in Figure 5:

- The user inserts his/her smart card and then selects a unique user identity $u_{id}$ and a user password $u_{pw}$ and inputs his biometric information $u_{bi}$. Then, the user randomly chooses an integer $u_{sk}\in Z_{q}^{*}$ as user private key and calculates his/her public key $u_{pk}=u_{sk}.p$.
- In addition, user Ui generates a random number r1 and a process to compute $bio_{i}=H\left(U_{Bi}⊕r_{1}\right)$ and calculates $m_{i}=H\left(u_{id}⊕u_{pw}⊕bio_{i}⊕r_{1}\right)$. The user Ui consequently encrypts the message with the AES private key $\epsilon_{k}\{u_{id}\|u_{pw}\|bio_{i}\left\|m_{i}\right\}$, and it encrypts the AES private key with the ECC shared public key Msg.1. $E_{pk}\{k_{p}\{u_{id}\|u_{pw}\|bio_{i}\|m_{i}\}\}$ and sends it to the AS in the CPS.
- Upon receiving the message, AS will use his private key to decrypt the AES key, $d_{s}\left\{\epsilon_{k}\left\{u_{id}⊕u_{pw}⊕bio_{i}⊕m_{i}\right\}\right\}$, and then it uses the AES key to obtain the user information $\mu_{k}\{u_{id}\|u_{pw}\|bio_{i}\left\|m_{i}\right\}$. The AS verifies the received information with the one in the database. If the user exists, the AS will notify the $U_{i}$ to choose another identity; otherwise, the AS computes $a_{i}=H\left(u_{id}\|s\right),F_{i}=H\left(bio_{i}\|s\right)$ and calculates $R_{i}=a_{i}⊕H\left(bio_{i}⊕m_{i}\right)$, $n_{i}=f_{i}⊕H\left(u_{id}⊕m_{i}\right)$ and $x_{i}=H(a_{i}\|f_{i}\left\|m_{i}\right)$.
- Next, the authentication server AS will embed the calculated parameters $\{x_{i},n_{i},r_{i},$H(.),q,p} onto the smart card SC and will send it to the $U_{i}$. Those parameters will also be stored in the database and recorded as an enrolled user. Now, AS sends the parameter to the $U_{i}$ via a secure channel.
- The user $U_{i}$ receives the embedded smart card SC and writes the parameters Msg.2: $\left\{x_{i},n_{i},r_{i},H\left(.\right),q,p\right\}$ into the smart card and stores $r_{1}$ in the memory.

### 5.3. Fog Node Registration Phase

**Figure 6:**
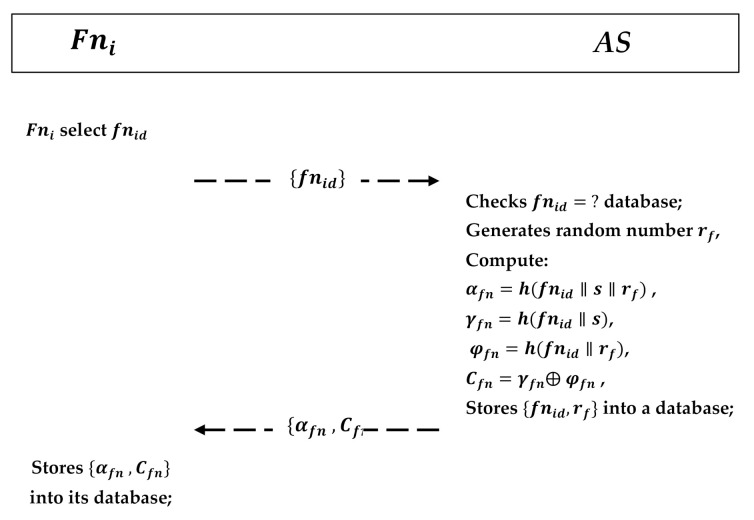
Fog node registration phase.

This phase requires the fog node $Fn_{i}$ to register itself into the CPS. As shown in Figure 6, a fog node $Fn_{i}$ performs the following steps:

- The $Fn_{i}$ firstly selects an identity $fn_{id}$ and sends $fn_{id}$ it to the AS in the CPS via a secure channel.
- AS receives it and checks whether the identity exists or not by comparing $fn_{id}=fn_{id}$ stored in the database; after verification, AS generates its own random number $r_{f}$; computes $\alpha_{fn}=h(fn_{id}\left\|s\right\|r_{f})$, $\gamma_{fn}=h\left(fn_{id}\|s\right)$, $\rho_{fn}=h\left(fn_{id}\|r_{f}\right)$, and $C_{fn}=\gamma_{fn}⊕\rho_{fn}$; stores $\left\{fn_{id},r_{f}\right\}$ into a database; and sends $\left\{\alpha_{fn},C_{fn}\right\}$ back to $Fn_{i}$ securely.
- $Fn_{i}$ receives $\alpha_{fn},C_{fn}$ and stores it in its database.

### 5.4. Login Phase

**Figure 7:**
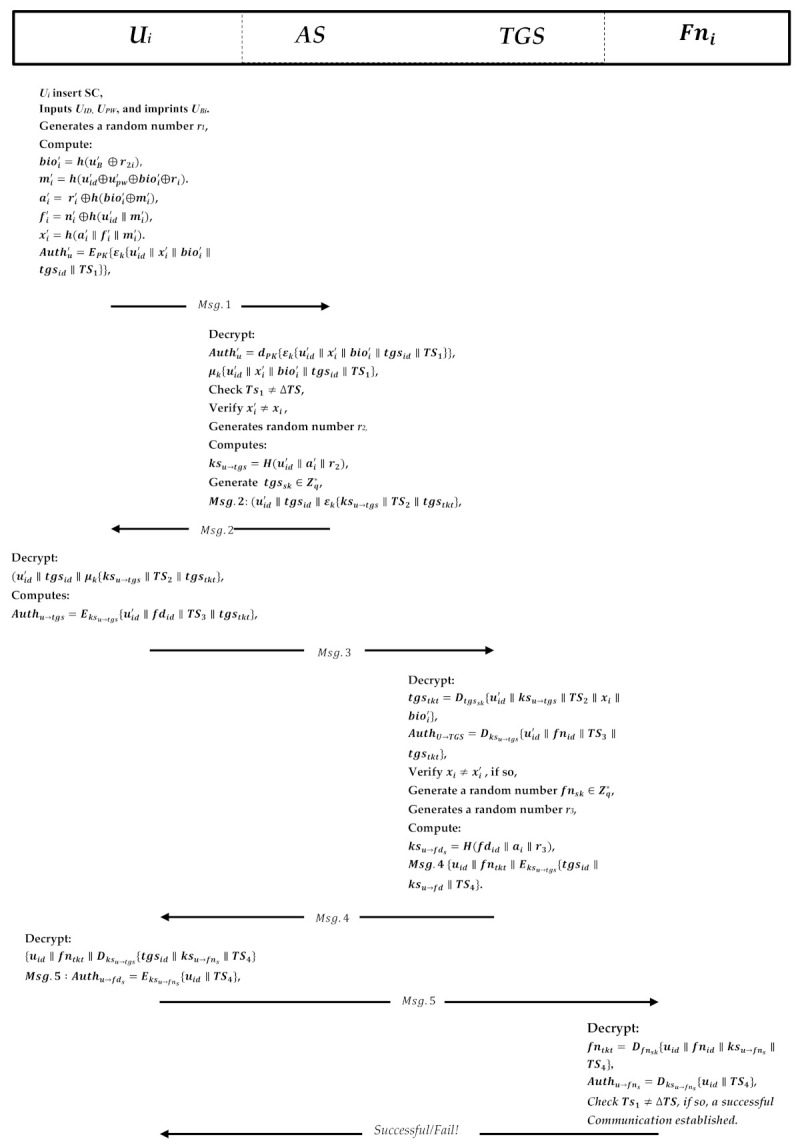
Login and authentication phase.

When user $U_{i}$ wants to access the data stored on the cross-platform fog server, she/he inserts her/his smart card into the terminal and performs the following steps to log into the system. Figure 7 shows the process of the login and authentication phases.

- The user $U_{i}$ inserts her/his identity $u_{id}$ and password $u_{pw}$; inputs his/her biometric information; and extracts the random number and the information stored in the smart card SC. $U_{i}$ computes $bio_{i}=h\left(u_{B}^{{}^{\prime }}⊕r_{2i}\right)$, $m_{i}^{{}^{\prime }}=h\left(u_{id}^{{}^{\prime }}⊕u_{pw}^{{}^{\prime }}⊕bio_{i}^{{}^{\prime }}⊕r_{i}\right).$
- Then, based on the stored parameters, it will calculate $a_{i}^{{}^{\prime }}=r_{i}^{{}^{\prime }}⊕h\left(bio_{i}^{{}^{\prime }}⊕m_{i}^{{}^{\prime }}\right)$, $f_{i}^{{}^{\prime }}=n_{i}^{{}^{\prime }}⊕h\left(u_{id}^{{}^{\prime }}\|m_{i}^{{}^{\prime }}\right)$, and $x_{i}^{{}^{\prime }}=h(a_{i}^{{}^{\prime }}\|f_{i}^{{}^{\prime }}\left\|m_{i}^{{}^{\prime }}\right)$.
- Next, the smart cart computes an authentication message encrypted with the AES private key and the ECC public key $Auth_{u}^{{}^{\prime }}=E_{PK}\{\xi_{k}\{u_{id}^{{}^{\prime }}\|x_{i}^{{}^{\prime }}\|bio_{i}^{{}^{\prime }}\|tgs_{id}\|TS_{1}\}\}$, where the TS is the current user timestamp. Then, U sends the Msg.1 to the AS.
- Upon receiving the authentication request message Msg.1, the server decrypts the message utilizing its private key to decrypt the message $Auth_{u}^{{}^{\prime }}=d_{PK}\{\epsilon_{k}\{u_{id}^{{}^{\prime }}\|x_{i}^{{}^{\prime }}\|bio_{i}^{{}^{\prime }}\|tgs_{id}\|TS_{1}\}\}$, and then the encrypted message with the AES key decrypts the parameters with the same key to obtain the information $\mu_{k}\{u_{id}^{{}^{\prime }}\|x_{i}^{{}^{\prime }}\|bio_{i}^{{}^{\prime }}\|tgs_{id}\left\|TS_{1}\right\}$.
- After that, AS checks the timestamp to see if it is similar to the server timestamp, extracts the $u_{id}^{\prime }$, and verifies $x_{i}^{\prime }=x_{i}$ whether it is valid or not; if not, the session is terminated. Otherwise, AS proceeds generating a random integer number $r_{2}$ and computes the key sessions known to protect the communication between the user and the ticket-granting service. It is only known to the $U_{i}$ and AS.
- Next, AS computes the key session $ks_{u\to tgs}=H(u_{id}^{{}^{\prime }}\|a_{i}^{{}^{\prime }}\left\|r_{2}\right)$ and generated another random number $tgs_{sk}\in Z_{q}^{*}$ as the TGS secret key is known to AS and TGS only. AS then forwardd the TGS secret key to the TGS along with $\langle X_{i}^{{}^{\prime }},Bio_{i}^{{}^{\prime }}\rangle$ to the TGS.
- Then, AS prepares the message Msg.2: $u_{id}^{{}^{\prime }}\|tgs_{id}\|\epsilon_{k}\{ks_{u\to tgs}\|TS_{2}\left\|tgs_{tkt}\right\}$, where $tgs_{tkt}=E_{tgs_{sk}}\{u_{id}^{{}^{\prime }}\|ks_{u\to tgs}\|TS_{2}\|x_{i}\left\|bio_{i}^{{}^{\prime }}\right\}$ is the Ticket. The $tgs_{tkt}$ cannot be decrypted but the TGS only. It then sends the messages Msg.2 to the user to enable her/him to authenticate the TGS.

### 5.5. Authentication Phase

In this phase, the user will decrypt the message A to obtain the critical session and the parameters after successfully receiving the messages from the authentication server. The message B will be forwarded to the TGS. The steps of the authentication phase are given below:

- The user decrypts message Msg.2 using the key $\mu_{k}$ to get the critical session $ks_{u\to tgs}$ and other information $(u_{id}^{{}^{\prime }}\|tgs_{id}\|\mu_{k}\{ks_{\left(u\to tgs\right)}\|TS_{2}\left\|tgs_{tkt}\right\}$, and contains the Ticket granting service ticket $tgs_{tkt}$ and this message is encrypted by the $tgs_{sk}$ and the user cannot modify the Ticket in private. Therefore, $U_{i}$ will forward it to the TGS as message Msg.3.
- It then computes an authentication message $Auth_{u\to tgs}=E_{{ks}_{u\to tgs}}\{u_{id}^{{}^{\prime }}\|fn_{id}\|TS_{3}\left\|tgs_{tkt}\right\}$, which contains the user identity, fog node identity, the current user timestamp, and the TGS ticket. The message is encrypted with the key session $ks_{u\to tgs}$ that is shared to communicate $U_{i}$ and TGS. The user sends a request to the TGS to get permission to visit the fog node server.
- Upon receiving the request from Ui, TGS decrypts the Ticket $tgs_{tkt}=D_{{tgs}_{sk}}\{u_{id}^{{}^{\prime }}\|ks_{u\to tgs}\|TS_{2}\|x_{i}\left\|bio_{i}^{{}^{\prime }}\right\}$ to obtain a key session and decrypts the authenticator message as well by using the shared key sessions $Auth_{u\to tgs}=D_{{ks}_{u\to tgs}}\{u_{id}^{{}^{\prime }}\left\|fn_{id}\right\|TS_{3}$$\|tgs_{tkt}\}$.
- Next, it verifies the $x_{i}\neq x_{i}^{{}^{\prime }}$ that was received earlier from AS; if it is not equal, the session will be terminated; if yes, then the TGS will generate a random number $fn_{sk}\in Z_{q}^{*}$ as the fog node secret key that will be known to the TGS and the fog node server, and it will then be sent to the fog node along with $<T_{4}>$.
- Then, it generates a random number r3 to compute the key session $ks_{u\to fn_{s}}=H(fn_{id}\|a_{i}\left\|r_{3}\right)$ and composes the message Msg.4 $\{u_{id}\|fn_{tkt}\|E_{ks_{u}\to tgs}\{tgs_{id}\|ks_{u\to fn}\|TS_{4}\}\}$, where the fog node ticket is $fn_{tkt}=E_{{fn}_{sk}}\{u_{id}\|fn_{id}\|ks_{u\to {fn}_{s}}\left\|TS_{4}\right\}$, which contains user the identity, fog node identity, shared key session, and the current timestamp. It then sends the message Msg.4 to the user to enable him/her to authenticate to the fog node.
- The user $U_{i}$ receives information from the TGS; it will firstly decrypt the message $\{u_{id}\|fn_{tkt}\|D_{{ks}_{u\to tgs}}\{tgs_{id}\|ks_{u\to fn_{s}}\|TS_{4}\}\}$ to get the shared session key, and the user cannot decrypt the fog node Ticket.
- Next, the user generates an authenticator message Msg.5: $Auth_{u\to {fn}_{s}}=E_{ks_{u\to {fn}_{s}}}\{u_{id}\|$$TS_{4}\}$, and composes the Ticket $fn_{tkt}=E_{{fn}_{sk}}\{u_{id}\|fn_{id}\|ks_{u\to {fn}_{s}}\left\|TS_{4}\right\}$. Then, it will send the messages to the fog node for mutual authentication.
- The fog node server receives the message Msg.5, and it will decrypt the message from the secret key that it shared earlier from the TGS $fn_{tkt}=D_{{fn}_{sk}}\{u_{id}\|fn_{id}\|ks_{u\to {fn}_{s}}\left\|TS_{4}\right\}$ to get a key session.
- Then, the server decrypts the authenticator message using a shared key session $Auth_{{u\to fn}_{s}}=D_{ks_{{u\to fn}_{s}}}\left\{u_{id}\|TS_{4}\right\}$, and checks the authenticator timestamp with a shared timestamp and if it is not equal, the server terminates the session; if yes, then the client can trust the server and can start issuing service requests to the server and send a successful message. The fog node server now provides the requested services to the user.

## 6. Security Analysis

The security analysis is carried out formally and informally in this section. The formal security analysis of the proposed scheme was conducted with the BAN logic (Burrows–Abadi–Needham), a formal model that aims to see how information exchange can be secured from eavesdropping. Informal security analysis ensures that, e.g., the proposed scheme prevents different kinds of known attacks. The following paragraphs provide details of the BAN logic.

### 6.1. Mutual Authentication Proof Using BAN Logic

We conducted a BAN logic analysis to verify the proposed scheme with secure mutual authentication. Table 3 specifies the BAN logic notations and postulates and describes the goals, assumptions, idealized version formulas, and confirms secure mutual authentication in the proposed scheme before performing a BAN logic analysis.

#### 6.1.1. Message Exchanges

This section illustrates an optimal way of the message exchanges for our proposed scheme.

1. **The authentication service exchanges:** Firstly, the messages in the scheme are exchanged between the user and the authentication server (AS), also called the ticket exchange. The user applies for the ticket TGT to communicate with TGS and the session key from the Cloud Provider Server (CPS). The user needs to input his/her user identity and password to log into the network. The user sends a request message U_AS_REQ, and AS responds U_AS_REP.
  (a) Ui → AS: U_AS_REQ $\left\{u_{id}^{\prime },x_{i}^{\prime },bio_{i}^{\prime },tgs_{id},ts_{1}\right\}$.
  (b) AS → Ui: U_AS_REP $(u_{id}^{\prime },tgs_{id},E_{u_{pk}}\left\{ks_{u\to tgs},ts_{2},tgs_{tkt}\right\}$.
2. **The authorization service exchanges:** It is the process of the message’s exchanges between the user and the TGS to get a ticket to communicate with the fog server. The user sends a request encrypted with a shared session key and for decryption as well. The TGS exchange consists of two messages: U_TGS_REQ and U_TGS_REP.
  (a) Ui→AS: U_TGS_REQ $E_{ks_{u\to tgs}}\left\{u_{id}^{{}^{\prime }},fn_{id},ts_{3},tgs_{tkt}\right\}$.
  (b) AS→Ui: $U\_TGS\_REP\{u_{id},fn_{tkt},E_{ks_{u\to tgs}}\left\{tgs_{id},ks_{{u\to fn}_{s}},ts_{4},\right\}\}$.
3. **The user/fog server exchange:** In this process, the user gets the Ticket and shared key session known to the user edge and fog node server fn. The user can now communicate to the fn using the received information and will consist of two messages: U_FN_REQ and U_FN_REP. U_FN_REP is only used when there is a need for two-way authentication, and the server wants to prove its identity to the client.
  (a) Ui → FN: U_FN_REQ $\left(E_{fn_{sk}}\left\{u_{id},fn_{id},ks_{{u\to fn}_{s}},ts_{4}\right\}\right)$.
  (b) FN → Ui: U_FN_REP (success/fail).

#### 6.1.2. Goals and Assumptions

Initial assumptions: Hence, the authentication goals are given as follows:

- *Goal 1:* Ui **believes** $Ui\overset{pk}{\to }AS$.
- *Goal 2:* FN **believes** $FN\overset{ks_{fn,tgs}}{\to }TGS$.
- *Goal 3:* Ui **believes** AS **Controls** $Ticket_{ui,fn}$.
- *Goal 4:* Ui **believes** TGS **controls** $ks_{ui,fn}$.
- *Goal 5:* Ui **believes** TGS **controls** $ks_{ui,fn}$.
- *Goal 6:* FN **believes** TGS **controls** $ks_{ui,fn}$.
- *Goal 7:* Ui **believes fresh** ($TS_{1}$).
- *Goal 8:* FN **believes fresh** ($TS_{2}$).
- *Goal 9:* Ui **believes fresh** ($TS_{3}$).

#### 6.1.3. BAN Logic Proof

Based on the logical assumptions, the authentication goals of the proposed scheme can be illustrated according to the BAN logic as follows:

1. According to the message meaning rule (Rule 1): Ui believes the PK is a shared public key between Ui and AS. In addition, the Ui sees that $Tiket_{U,TGS}$ is encrypted with $ks_{u-tgs}$, and then Ui believes that AS once said $Ticket_{u,tgs}$.
  $$\frac{Ui\;\mathrm{believes}\;FN\overset{pk}{\to }TGS,Ui\;\mathrm{sees}\;{\left\{Ticket_{u,tgs}\right\}}_{ks_{ui\to tgs}}}{Ui\;\mathrm{believes}\;AS\;\mathrm{said}\;Ticket_{ui,tgs}}$$
2. By timestamp-verification rule (Rule 2): The Ui believes that [TS1] is fresh if Ui believes that AS once said $Ticket_{ui,tgs}$. Meanwhile, Ui believes that AS believes $Ticket_{u,tgs}$.
  $$\frac{Ui\;\mathrm{believes}\mathrm{fresh}(\mathrm{TS}1),\;Ui\;\mathrm{believes}\;AS\;\mathrm{said}\;Ticket_{ui,tgs}}{Ui\;\mathrm{believes}\;AS\;\mathrm{believes}\;Ticket_{ui,tgs}}$$
3. By Rule 3: If Ui believes that AS controls $Ticket_{ui,tgs}$, then Ui believes that AS believes $Ticket_{ui,tgs}$ and the Ui believes the $Ticket_{ui,tgs}$.
  $$\frac{Ui\;\mathrm{believes}\;AS\;\mathrm{controls}\;Ticket_{ui,tgs},\;Ui\;\mathrm{believes}\;AS\;\mathrm{believes}\;Ticket_{ui,tgs}}{Ui\;\mathrm{believes}\;Ticket_{ui,tgs}}$$
4. By Rule $4^{u}$,
  $$Ui\;\mathrm{believes}\;ks_{ui,tgs}$$
5. Again, by message meaning rule: Ui believes that $ks_{ui,tgs}$ is shared session key with TGS, and Ui sees the $Ticket_{ui,fn}$ is encrypted with $KS_{ui,tgs}$, and then Ui believes that TGS once said $Ticket_{ui,fn}$.
  $$\frac{Ui\;\mathrm{believes}\;Ui\overset{ks_{ui,tgs}}{\to }TGS,Ui\;\mathrm{sees}\;{\left\{Ticket_{ui,fn}\right\}}_{ks_{ui\to tgs}}}{Ui\;\mathrm{believes}\;TGS\;\mathrm{believes}\;Ticket_{ui,fn}}$$
6. By Rule 2: Ui believes that the $\left(Ticket_{ui,fn}\right)$ is fresh, and Ui believes that TGS once said $Ticket_{ui,fn}$, while Ui believes that TGS believes $Ticket_{ui,fn}$.
  $$\frac{Ui\;\mathrm{believes}\mathrm{fresh}\;\;\left(Ticket_{ui,fn}\right),Ui\;\mathrm{believes}\;TGS\;\mathrm{said}\;Ticket_{ui,fn}}{Ui\;\mathrm{believes}\;TGS\;\mathrm{believes}\;Ticket_{ui,fn}}$$
7. Again, by Rule 3, if Ui believes that TGS controls $Ticket_{ui,fn}$, then Ui believes that TGS believes $Ticket_{ui,fn}$ and Ui believes $Ticket_{ui,fn}$.
  $$\frac{Ui\;\mathrm{believes}\;TGS\;\mathrm{controls}\;Ticket_{ui,fn},Ui\;\mathrm{believes}\;TGS\;\mathrm{believes}\;Ticket_{ui,fn}}{Ui\;\mathrm{believes}\;\;Ticket_{ui,fn}}$$
8. Finally, according to Rule 4u
  $$Ui\;\mathrm{believes}\;Ui\overset{ks_{ui,fn}}{\to }FN$$
9. According to the message meaning rule: FN believes that $ks_{fn,tgs}$ is a shared session key with TGS, and FN sees the $\left\{Ticket_{ui,fn}\right\}$ is encrypted with $ks_{fn,tgs}$, and then FN believes that TGS once said $Ticket_{ui,fn}$.
  $$\frac{FN\;\mathrm{believes}\;FN\overset{ks_{fn,tgs}}{\to }TGS,FN\;\mathrm{sees}\;{\left\{Ticket_{ui,fn}\right\}}_{ks_{fn,tgs}}}{FN\;\mathrm{believes}\;TGS\;\mathrm{said}\;Ticket_{ui,fn}}$$
10. Again, by the timestamp-verification rule: The FN believes that [TS] is fresh if FN believes that TGS once said $Ticket_{ui,fn}$. Then, FN believes that TGS believes $Ticket_{ui,fn}$.
  $$\frac{FN\;\mathrm{believes}\mathrm{fresh}\;\left(TS\right),FN\;\mathrm{believes}\;TGS\;\;\mathrm{said}\;{\left\{Ticket_{ui,fn}\right\}}_{ks_{fn,tgs}}}{FN\;\mathrm{believes}\;TGS\;\mathrm{believes}\;Ticket_{ui,fn}}$$
11. By Jurisdiction rule again: If FN believes that TGS has jurisdiction over $Ticket_{ui,fn}$ and FN believes that TGS believes $Ticket_{ui,fn}$, then FN believes $Ticket_{ui,fn}$.
  $$\frac{FN\;\mathrm{believes}\;TGS\;\mathrm{controls}\;Ticket_{ui,fn},FN\;\mathrm{believes}\;TGS\;\mathrm{believes}\;Ticket_{ui,fn}}{FN\;\mathrm{believes}\;\;Ticket_{ui,fn}}$$
12. Finally, according to Rule $4^{u}$,
  $$FN\;\mathrm{believes}\;Ui\overset{ks_{ui,fn}}{\to }FN$$
13. By Rule 1 (message meaning rule): If FN believes that the $\left(ks_{ui,fn}\right)$ is a shared session key with Ui, then, FN sees that $TS_{3}$, $Ui\overset{ks_{ui,fn}}{\to }FN$ is encrypted with $ks_{ui,fn}$, and FN believes that Ui once said $Ui\overset{ks_{ui,fn}}{\to }FN$.
  $$\frac{FN\;\mathrm{believes}\;Ui\overset{ks_{ui,fn}}{\to }FN,FN\;\mathrm{sees}\;{\left\{TS_{3},Ui\overset{ks_{ui,fn}}{\to }FN\right\}}_{ks_{ui,fn}}\;}{FN\;\mathrm{believes}\;Ui\;\mathrm{said}\;Ui\overset{ks_{ui,fn}}{\to }\;FN}$$
14. By Rule 2: If FN believes that $Ui\overset{ks_{ui,fn}}{\to }FN$ is fresh and FN believes that Ui once said $Ui\overset{ks_{ui,fn}}{\to }FN$, then FN believes that Ui believes $Ui\overset{ks_{ui,fn}}{\to }FN$.
  $$\frac{FN\;\mathrm{believes}\mathrm{fresh}\;\left(Ui\overset{ks_{ui,fn}}{\to }FN\right),FN\;\mathrm{believes}\;Ui\;\mathrm{said}\;Ui\overset{ks_{ui,fn}}{\to }FN}{FN\;\mathrm{believes}\;Ui\;\mathrm{believes}\;Ui\overset{ks_{ui,fn}}{\to }\;FN}$$
  Finally, we derive that FN believes that Ui believes.
  $$FN\;\mathrm{believes}\;Ui\;\mathrm{believes}\;Ui\;\overset{ks_{ui,fn}}{\to }\;FN.$$
  Similarly, we can get,
  $$Ui\;\mathrm{believes}\;FN\;\mathrm{believes}\;Ui\overset{ks_{ui,fn}}{\to }\;FN.$$
  The above demonstrates our authentication goal and proves that this scheme ensures that the user and the fog node are mutually communicated.

### 6.2. Informal Security Analysis

This section illustrates several security problems and shows that the proposed scheme is secure from various types of malicious attacks as follows:

**Theorem** **1.** *The proposed scheme avoids the key escrow problem inherited by the Identity based cryptography (IBC).*

**Proof** **of** **Theorem** **1.** As mentioned above, a distinctive user identity is assigned as $u_{id}$ and biometric $u_{bi}$, while the assigned secret key is $u_{pk}$. Please note that $u_{pk}$ is stored in the SC record and shared between the user and the fog server. Subsequently, the user itself randomly generates its secret key $u_{sk}\in Z_{q}^{*}$, which will later be kept a secret to AS. The secret key is generated based on the random number $r_{1}$, and the server has no access to it. In this way, other entities cannot extract $r_{1}$ from the published $bio_{i}=H\left(u_{bi}⊕r_{1}\right)$ or $m_{i}=H\left(u_{id}⊕u_{pw}⊕bio_{i}⊕r_{1}\right)$. Similarly, the authentication request is encrypted with the user public key. □

**Theorem** **2.** *The proposed scheme is secure from a replay attack.*

**Proof** **of** **Theorem** **2.** Assume that an adversary tries replaying the previously captured valid login and authentication messages $<u_{id}^{\prime },x_{i}^{\prime },bio_{i}^{\prime },ts_{1}>$. The message is encrypted by using the server public key PK, and it takes a fresh timestamp $ts_{n}$ to validate a legitimate user. After the server decrypts the message to obtain user information, AS will verify the received timestamp TS1 with the server’s current stamp TS▵, $TS_{1}\neq TS▵$. Likewise, if the adversary tries to replay the authentication message by replaying $TS_{1}$ with $TS_{2}$, it will not be able to pass because $X_{i}^{\prime }$ is encrypted using a one-way hash function $x_{i}^{{}^{\prime }}=H(a_{i}^{{}^{\prime }}\|f_{i}^{{}^{\prime }}\left\|m_{i}^{{}^{\prime }}\right)$ so, the AS can detect any changes in the message. Hence, the scheme is resistant to a replay attack. □

**Theorem** **3.** *The proposed scheme is secure from the impersonation attack.*

**Proof** **of** **Theorem** **3.** The adversary in this attack is trying to provide a login message by eavesdropping or computing a message to deceive the AS as a legal user. In the proposed scheme, if the adversary tries to replay the previous message or to impersonate $<u_{id}^{{}^{\prime }},x_{i}^{{}^{\prime }},bio_{i}^{{}^{\prime }},ts_{1}>$, the AS will validate the message by checking $x_{i}^{{}^{\prime }}\neq x_{i}$. Moreover, the adversary cannot capture the valid $x_{i}^{\prime }$, due to the lack of the user identity $u_{id}^{\prime }$, user password $u_{pw}^{\prime }$ and user biometric info $u_{bi}^{\prime }$. Therefore, a malicious user cannot impersonate a legitimate user to access the fog node. □

**Theorem** **4.** *The proposed scheme is resistant to a man-in-the-middle attack.*

**Proof** **of** **Theorem** **4.** Assume that the adversary intercepts the login and authentication messages successfully $\left\{u_{id}^{{}^{\prime }}\|x_{i}^{{}^{\prime }}\|bio_{i}^{{}^{\prime }}\|tgs_{id}\|ts_{1}\right\}$, $\left\{u_{id}^{{}^{\prime }}\|ks_{u\to tgs}\|ts_{2}\|x_{i}\|bio_{i}^{{}^{\prime }}\right\}$, and $\left\{u_{id}^{{}^{\prime }}\|fn_{id}\|ts_{3}\|tgs_{tkt}\right\}$. The adversary will fail because there is a crucial session KS established between all the entities and shared after the mutual authentication is generated between them. In addition, the ticket-granting service is sharing the encrypted ticket $tgs_{tkt}=E_{{tgs}_{sk}}\left\{u_{id}^{{}^{\prime }}\|ks_{u\to tgs}\|ts_{2}\left\|x_{i}\right\|bio_{i}^{{}^{\prime }}\right\}$ using the TGS secret key and only can only be decrypted by it, which goes for the communication between TGS and FN. However, for the same reason mentioned above, the attacker cannot pass this process without knowing the patient’s uid and the personal values $x_{i}$ and TS. Therefore, the adversary cannot cheat the user Ui to share a key session and believe that the key is shared with the authentication server AS, and this judgment also works on the FN. Therefore, the adversary cannot launch the man-in-middle attack successfully to cheat either the user or the servers in the proposed scheme. □

**Theorem** **5.** *The proposed scheme withstands the known-key attacks.*

**Proof** **of** **Theorem** **5.** The proposed scheme provides resistance against known-key session attacks according to the unique key session that has been generated between each entity. The key session in the proposed scheme is calculated based on $<a_{i}^{{}^{\prime }}\|r_{2}>$ that makes it unique because the random integer *r* is generated randomly and independently by the Ui, AS, and TGS. Since the $r_{1}$, $r_{2}$, and $r_{3}$ are different from each other, the critical session in each run is unique in the proposed scheme. Therefore, the proposed scheme is resisting this attack. Using a unique key session during every communication session allows achieving freshness of the key in the proposed scheme. The session keys are generated in AS $ks_{u\to tgs}=H(u_{id}^{{}^{\prime }}\|a_{i}^{{}^{\prime }}\left\|r_{2}\right)$ and TGS $ks_{u\to {fn}_{S}}=H(fn_{id}\|a_{i}\left\|r_{3}\right)$, differently and independently. □

**Theorem** **6.** *The proposed scheme withstands privileged insider attacks.*

**Proof** **of** **Theorem** **6.** Assume that the adversary attempts to obtain the legal user information $u_{id}$ and $u_{pw}$, but he/she will fail to impersonate since he/she must provide the correct biometrics $u_{bi}$ of the targeting user. In addition, it will be difficult for the attacker to obtain the legal user information since the proposed scheme is performing a hash function on the user information $H(u_{id}⊕u_{pw}⊕bio_{i}⊕r_{1})$ and contains a randomly generated number in it. The user biometric is protected as well in the hash formatting with the random number $r_{1}$. The attacker cannot extract the stored parameters from the stored hash value successfully; thus, the proposed scheme works against any insider attack. □

**Theorem** **7.** *The proposed scheme withstands a stolen smart card attack.*

**Proof** **of** **Theorem** **7.** In this attack, the adversary attempts to extract the user information stored in the smart card. He/she will fail since the parameters $\left\{x_{i},n_{i},R_{i},H\left(.\right),q,p\right\}$ are secured and the attacker cannot successfully compute $H(a_{i}\|f_{i}\left\|m_{i}\right)=x_{i},f_{i}⊕H\left(u_{id}\|m_{i}\right)=n_{i}$, and $\;a_{i}⊕H\left(bio_{i}\|m_{i}\right)=R_{i}$ as they are secured using a collision-resistant one-way hash function H(.). Therefore, the attacker is unable to determine the user information $u_{id}$ and $u_{pw}$. Therefore, the proposed scheme is resistant to the stolen smart card attack. □

**Theorem** **8.** *The proposed scheme is secure from a replay attack.*

**Proof** **of** **Theorem** **8.** The proposed scheme is resistant to a server spoofing attack. An adversary exploits a legitimate user’s information to counterfeit as a server. To successfully impersonate as an authentication server AS, it cannot compute the $(u_{id}^{{}^{\prime }}\|tgs_{id}\|E_{u_{pk}}\{ks_{u\to tgs}\|ts_{2}\left\|tgs_{tkt}\right\}$, and $tgs_{tkt}=E_{{tgs}_{sk}}\{u_{id}^{{}^{\prime }}\|ks_{u\to tgs}\|ts_{2}\|x_{i}\left\|bio_{i}^{{}^{\prime }}\right\}$; to compute the correct values, a malicious server needs to know the critical session KS, timestamp TS, and the secret value Xi. The values are encrypted and cannot be decrypted by only using the secret server key that is shared earlier. As mentioned above, he/she will need to know the user identity u−id to compute critical sessions. □

**Theorem** **9.** *The proposed scheme withstands a Denial of Service (DoS) Attack.*

**Proof** **of** **Theorem** **9.** This attack can suspend services of the server by flooding the network, but the proposed scheme is resistant to this attack. Since the proposed scheme verifies the user identity $u_{id}$ and password $u_{pw}$, it also verifies the secret value $X_{i}^{{}^{\prime }}\neq X_{i}$ so he/she fails. Moreover, the server will detect a false message sent to it by the adversary using the timestamp (TS1, TS2, and TS3). The authentication server AS and FN will only proceed if the login message passes the check $(TS_{1}-T_{curr})\leq ▵TS$, and the Ticket granting server $(TS_{2}-T_{curr})\leq ▵TS$. The FN will also not process the message only if the shared timestamp matches the shared timestamp TS3. In addition, the TGS will check the validity of the $X_{i}^{{}^{\prime }}\neq X_{i}$ after the AS. □

**Theorem** **10.** *The proposed scheme is secure from an offline password guessing attack.*

**Proof** **of** **Theorem** **10.** The user identity $u_{id}$, password $u_{pw}$, and the biometric $u_{bi}$ are secured using a one-way hash function $H\left(u_{bi}⊕R_{i}\right)=bio_{i},H\left(u_{id}⊕u_{pw}⊕bio_{i}⊕r_{1}\right)=m_{i}$ and contains a random number in it. In addition, any alteration in the login message will be detected after the server verification. The attacker can never validate the password with a stolen smart card SC. If the attacker intercepts the login message SC $\left\{X_{i},n_{i},R_{i},H\left(.\right),q,p\right\}$ as a legitimate user, he/she will need to guess the user identity and password that server will validate the message after decrypting. Thus, the proposed scheme is highly secured against offline password guessing attacks. □

**Theorem** **11.** *The proposed scheme facilitates user anonymity.*

**Proof** **of** **Theorem** **11.** Assume an adversary intercepts the message $\left\{u_{id}^{{}^{\prime }}\|x_{i}^{{}^{\prime }}\|bio_{i}^{{}^{\prime }}\|tgs_{id}\|ts_{1}\right\}$. The attacker cannot obtain the information because the authentication message is encrypted using the authentication server public key $E_{PK}\left\{\right\}$. In addition, the server will check the validity of the user by extracting the original user’s identity $u_{id}$ and the secret value $x_{i}^{{}^{\prime }}\neq x_{i}$ as well. The AS and TGS will generate a key session using a unique random number with every communication session. Moreover, the adversary cannot launch a guessing attack to obtain the user information, because with knowledge of $x_{i}$, an adversary cannot compute $H\left(u_{id}⊕u_{pw}⊕bio_{i}⊕r_{1}\right)=m_{i}$ successfully according to the using of a one-way hash function as well. Therefore, nobody will be able to know the real identity of the user, except the user himself and the server. □

**Theorem** **12.** *The proposed scheme facilitates against user traceability attacks.*

**Proof** **of** **Theorem** **12.** The proposed scheme protects the real user identity, and the transmitted message is changed by updating r during every session. The transmitted messages are different from one session to the other since there is a new key session. KS is computed based on a new random number when every new session begins. Therefore, the attacker cannot distinguish whether the intercepted messages belong to the same user or not. Therefore, the proposed scheme provides user untraceability. □

**Theorem** **13.** *The proposed scheme facilitates a mutual authentication property.*

**Proof** **of** **Theorem** **13.** The authentication scheme needs to allow all the considered entities in communication to verify the identity of each other mutually. The use of ECC is in providing mutual authentication. The user and the server can authenticate each other by checking session key freshness $ks=H(u_{id}^{{}^{\prime }}\|a_{i}^{{}^{\prime }}\|r)$ in every session, and verifying the $x_{i}\neq x_{i}^{{}^{\prime }}$ with the timestamp, respectively, in the AS and the TGS. Therefore, the proposed scheme achieves mutual authentication. □

**Theorem** **14.** *The proposed scheme achieves perfect forward secrecy property.*

**Proof** **of** **Theorem** **14.** In the proposed scheme, the adversary cannot generate the key session $ks_{u\to tgs}=H(u_{id}^{{}^{\prime }}\|a_{i}^{{}^{\prime }}\left\|r_{2}\right)$, $ks_{u\to {fn}_{S}}=H(fn_{id}\|a_{i}\left\|r_{3}\right)$ because the adversary does not know the user identity. Therefore, the attacker cannot obtain the user/server identity. To successfully compute the key session, the attacker needs a secret value $A_{i}^{{}^{\prime }}$, and random number r, but he/she will fail because it is impossible to obtain the random number and the value $A_{i}^{{}^{\prime }}$ is secured using the one-way hash function $a_{i}^{{}^{\prime }}=R_{i}^{{}^{\prime }}⊕H\left(bio_{i}^{{}^{\prime }}\|m_{i}^{{}^{\prime }}\right)$ In addition, it includes protected biometric. As a result, it is difficult to determine the information, and the proposed scheme is achieving forward secrecy. □

**Theorem** **15.** *The proposed scheme achieves a biometric protection property.*

**Proof** **of** **Theorem** **15.** The user biometric $u_{bi}^{{}^{\prime }}$ is highly protected by a high entropy random number integer r and one-way hash function $bio_{i}^{{}^{\prime }}=H\left(u_{bi}^{{}^{\prime }}⊕r_{1}\right)$. Assume the adversary obtains the stored information on the smart card; but he/she cannot extract the User biometric $u_{bi}^{{}^{\prime }}$ without the knowledge of the user identity $u_{id}^{}{}^{\prime }$ and password $u_{pw}^{{}^{\prime }}$. Therefore, the proposed scheme of protects the user’s biometric. □

## 7. Formal Security Verification Using AVISPA Tool: Simulation Study

Simulations were carried out to test the proposed security framework using AVISPA [39], an extensively used security analysis model. It proves that the scheme avoids replay and man-in-the-middle attacks. This section includes a simple overview of the AVISPA tool [40]. It then shows the implementation code for the User (U), authentication server (AS), ticket-granting server (TGS), fog node (FN), session, goals, and the environment in High-Level Protocol Specification Language (HLPSL). Third, the simulation results are demonstrated.

### 7.1. AVISPA Tool Basic Explanation

AVISPA is a simulation verification tool to validate authentication schemes. Specification language (HLPSL) is used to implement the simulation code. AVISPA is a participant-related program. Each participant is autonomous and has some knowledge across channels in the form of communication parameters. First, wrote the code into HLPSL in this tool, and then used hlpsl2if to translate it into an intermediate (IF) format. AVISPA is currently being introduced in four back ends: (a) CL-AtSe; (b) OFMC; (c) SATMC; and (d) TA4SP. AVISPA is implemented on a backend basis on-the-fly model checker (OFMC); the output format is generated and then represented based on these back-ends, confirming that the system is safe from active and passive attacks.

### 7.2. Discussion of Proposed Scheme in HLPSL

**Figure 8:**
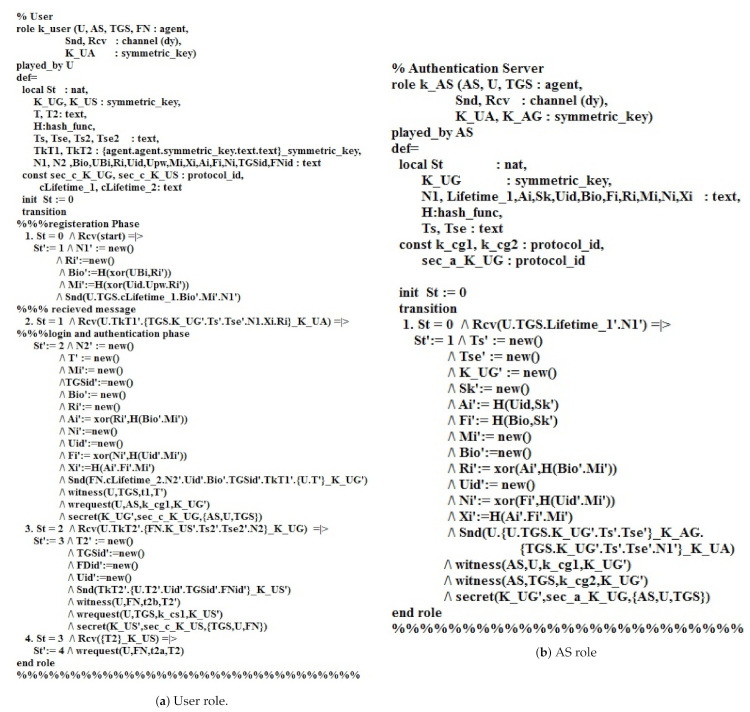
The User and AS roles in HLPSL.

The role of user U in HLPSL is shown in Figure 8a. In the registration phase, U sends $\left\{u_{id},u_{pw},bio_{i},m_{i}\right\}$ to the authentication server AS using Snd() operation via a secure channel. The declaration channel (dy) is made for the Dolev–Yao threat model. Accordingly, two declaration secrets, i.e., $\left(K\_UG^{\prime },sec\_c\_K\_UG,AS,U,TGS\right)$, $\left(K\_US^{\prime },sec\_c\_K\_US,TGS,U,S\right)$ state that $Bio,u_{pw}$ is only known to U, D_S is only known by AS and U_ID is known to U, AS, and TGS. After that, U obtains the smart card having the values, i.e., x_i,N_i,R_i, from AS. In the login phase, the user further creates $N2^{\prime },T^{\prime },Mi^{\prime },TGSid^{\prime },Ri^{\prime },Ni^{\prime },Uid^{\prime }$ using a new (-) operation and transmitting $(Uid^{{}^{\prime }}.N2^{\prime }.$$Uid^{\prime }.Bio^{\prime }.TGSid^{\prime }.U.T^{\prime }\_K\_UG^{\prime })$ to the AS via a public channel. The declaration witness $\left(U,TGS,t1,T^{\prime }\right)$ tells that U creates T for AS. In the authentication phase, U gets a reply message $\left(U.TkT2^{\prime }.S.K\_US^{\prime }.Uid^{\prime }.Bio^{\prime }.Ts2^{\prime }.Xi^{\prime }.Tse2^{\prime }.N2\_K\_UG\right)$ from AS by using Rcv () operation. Further, the user creates $T2^{\prime },TGSid^{\prime },FNid^{\prime },Uid^{\prime }$ and transmits the message $Snd(TkT2^{\prime }.U.T2^{\prime }.Uid^{\prime }.TGSid^{\prime }.FNid^{\prime }\_K\_US^{\prime })$ to the TGS. The declaration request $\left(U,TGS,k\_cs1,K\_US^{\prime }\right)$ states that the user sends a request to the TGS for knowing K_US’. The declaration secret $\left(K\_US^{\prime },sec\_c\_K\_US,TGS,U,S\right)$ states that T is known to U, AS, and TGS. The user later receives a message $\left(Uid^{\prime }.TkT1^{\prime }T2\_K\_US\right)$ from the TGS. The user then messages to the fog node $\left(TkT2^{\prime }.U.T2^{\prime }.Uid^{\prime }.FNid^{\prime }\_K\_US^{\prime }\right)$. The declaration request (U, S, t2a, T2) states that the user sends a request to the fog node.

Figure 8b depicts the role of the authentication server AS in HLPSL. In the login phase, AS receives $\left(U.TGS.Lifetime_{1}^{{}^{\prime }}.N1^{{}^{\prime }}.Uid^{{}^{\prime }}.Bio^{{}^{\prime }}.TGSid^{{}^{\prime }}.Xi^{{}^{\prime }}\right)$ from the user. Then, AS create $\left(Ts^{\prime },Tse^{\prime },K\_UG^{\prime },Sk^{\prime },Mi^{\prime },Bio^{\prime },Uid^{\prime }\right)$ and sends $\left(TkT2^{\prime }.U.T2^{\prime }.Uid^{\prime }.TGSid^{\prime }\_K\_US^{\prime }\right)$ to the user. Moreover, the declaration witness $\left(AS,U,k\_cg1,K\_UG^{\prime }\right)$ and (AS,TGS,$k\_cg2,K\_UG^{\prime })$ indicates that AS generates a symmetric key for user U and TGS. Furthermore, the declaration secret $\left(K\_UG^{\prime },sec\_a\_K\_UG,AS,U,TGS\right)$ states that AS,U,TGS knows the value of $K\_UG^{\prime }$.

**Figure 9:**
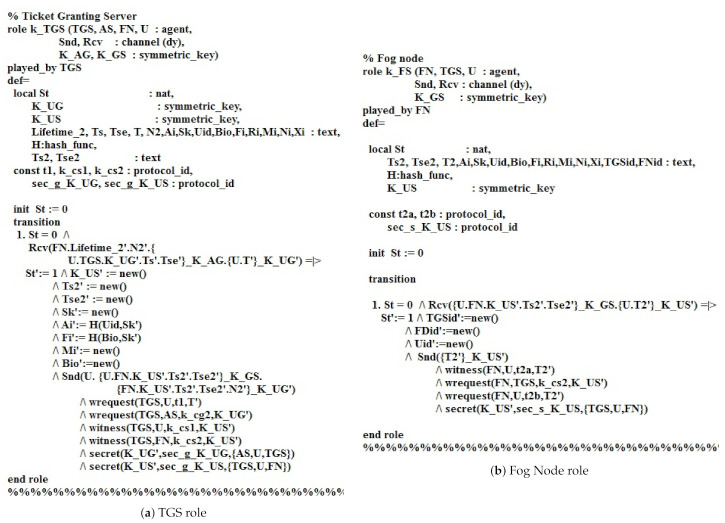
The TGS and Fog node roles in HLPSL.

Figure 9a presents the role of the Ticket granting server TGS in HLPSL. The role starts from the authentication phase, TGS receives $(S.Lifetime_{2}^{{}^{\prime }}.N2^{{}^{\prime }}.U.TGS.K\_UG^{{}^{\prime }}.$$Ts^{{}^{\prime }}.Uid^{{}^{\prime }},Xi^{{}^{\prime }},$$Bio^{{}^{\prime }}\_K\_AG.U.TkT2{}^{\prime }\_K\_UG^{\prime })$. Then, TGS transmits $U.U.S.K\_US^{\prime }.Ts2^{\prime }.Uid^{\prime },$$FNid^{\prime },Xi^{\prime }\_K\_$$GS.S.K\_US^{\prime }.Ts2^{\prime }Uid^{\prime },FNid^{\prime },Xi^{\prime }.Tse2^{\prime }.N2^{\prime }\_K\_UG^{\prime })$ to the user U. The request declaration $\left(TGS,U,t1,T^{\prime }\right)$ states that the user U sends a request to TGS for knowing $T^{\prime }$, where the request $\left(TGS,AS,k\_cg2,K\_UG^{\prime }\right)$ states that AS sends a request to TGS for knowing the $K\_UG^{\prime }$. The declaration witness $\left(TGS,U,k\_cs1,K\_US^{\prime }\right)$ indicates that the TGS generates $K\_US^{\prime }$ for U, where the declaration witness $\left(TGS,S,k\_cs2,K\_US^{\prime }\right)$ specifies that TGS generates $K\_US^{\prime }$ for the fog node server FN. Furthermore, the declaration secret $\left(K\_UG^{\prime },sec\_g\_K\_UG,AS,U,TGS\right)$ states that AS,U,TGS know the $K\_UG^{\prime }$, while the $\left(K\_US^{\prime },sec\_g\_K\_US,TGS,U,S\right)$ states that TGS,U,S know the value $K\_US^{\prime }$.

**Figure 10:**
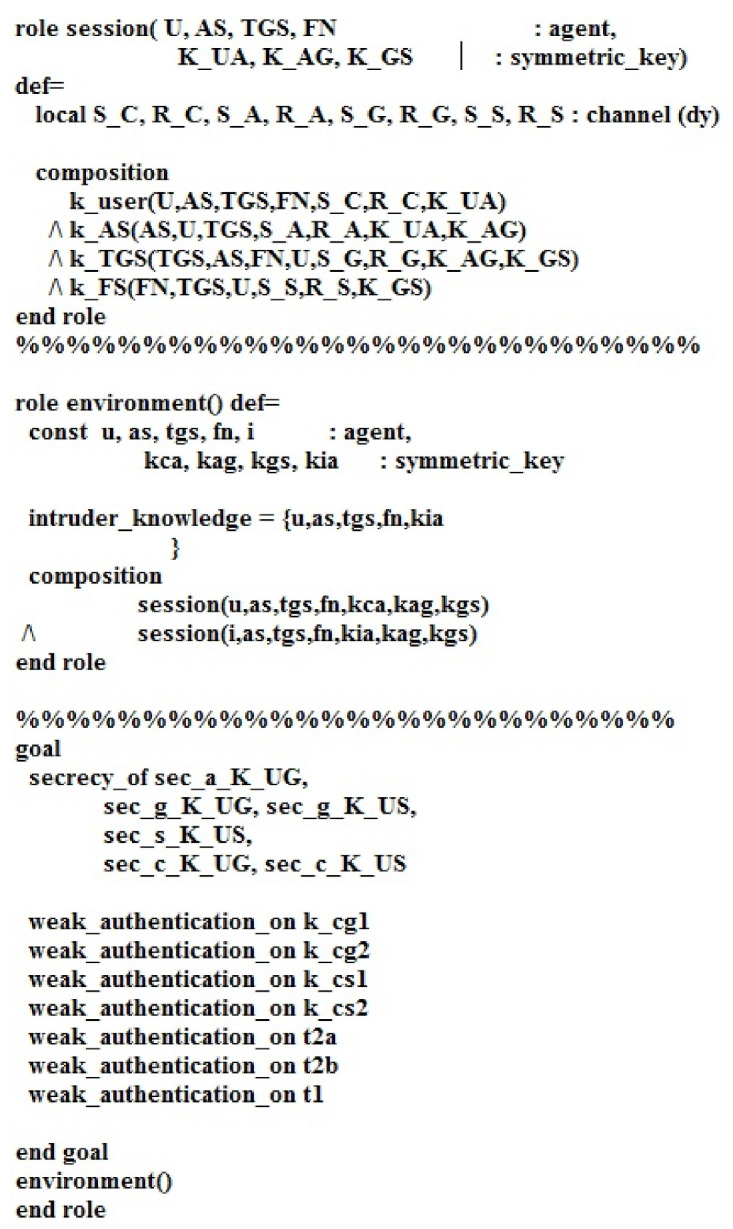
The session, goals, and environment roles in HLPSL.

Figure 9b depicts the role of the fog node FN in HLPSL. FN receives the message $\left(U.FN.K\_US{}^{\prime }.Ts2{}^{\prime }.Tse2{}^{\prime }.Uid{}^{\prime },FNid{}^{\prime },Xi{}^{\prime }\_K\_GS.U.T2{}^{\prime }\_K\_US{}^{\prime }\right)$ from the user. Then, FN provides the user message $\left(T2^{\prime }\_K\_US{}^{\prime }\right)$ and transmits it to the user. The declaration witness $\left(FN,U,t2a,T2^{\prime }\right)$ indicates that FN generates text T (Fail/Success) for the user. The declaration request $\left(FN,TGS,k\_cs2,K\_US^{\prime }\right)$ states that TGS sends a request to FN for knowing $K\_US^{\prime }$, where the declaration request $\left(FN,U,t2b,T2^{\prime }\right)$ states that the user sends a request to S for knowing the feedback of the fog node (Fail/Success). Moreover, the declaration secret (K_US′,sec_K_US,TGS,U,FN) states that $K\_US^{\prime }$ is known to TGS, U, and FN. Figure 10 demonstrates the roles of session, goals, and environment in HLPSL.

**Goals:**

- sec_a_K_UG: It tells that the AS, U, and TGS know K_UG’.
- sec_g_K_UG: It states that K_UG’ is shared among TGS, U, and FN.
- sec_c_K_UG: It shows that AS, U, and TGS know the value K_UG’.
- sec_c_K_US: It tells that the TGS, U, and FN know the K_US’.
- sec_g_K_UG: It states that the K_UG’ is shared among AS, U, and TGS.
- sec_g_K_US: It tells that TGS, U, and FN know the value K_US’.

**Authentications:**

- authentication_on k_cg1: The Ticket is only shared between the user and the TGS.
- authentication_on k_cg2: The timestamp is only valid for the User to the TGS authentication session.
- authentication_on k_cs1: The user sends the second ticket is only known by the User and the FN.
- authentication_on k_cs2: The timestamp is valid only for the authentication session to FN.
- authentication_on t2a: The first TS is replaced with a fresh one between the User and the TGS.
- authentication_on t2b: The User generates a fresh timestamp to authenticate to the FN.
- authentication_on t1: FN replies a text to the user about successful or failed authentication.

### 7.3. Simulation Results

**Figure 11:**
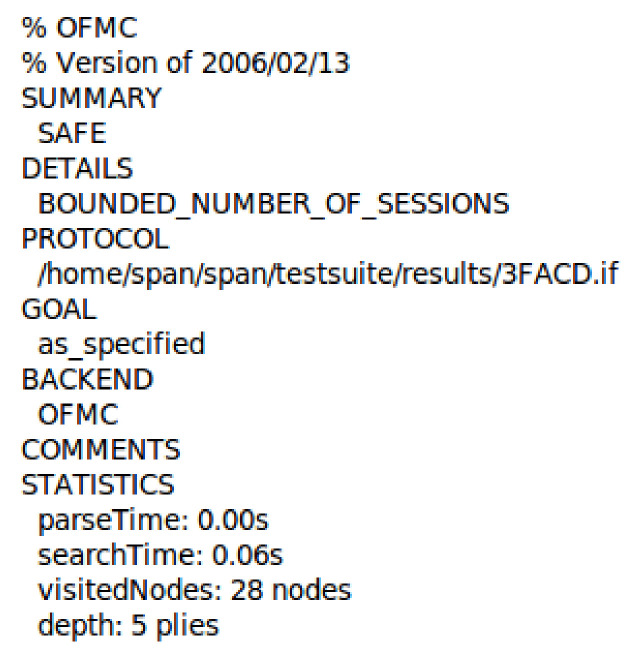
The simulation result using OFMC back-end.

**Figure 12:**
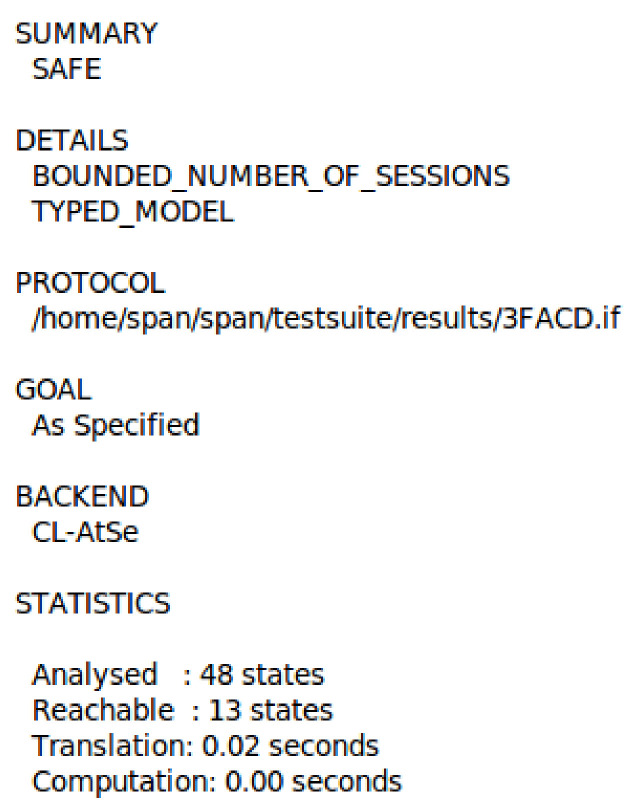
The simulation result using CL-AtSe back-end.

**Figure 13:**
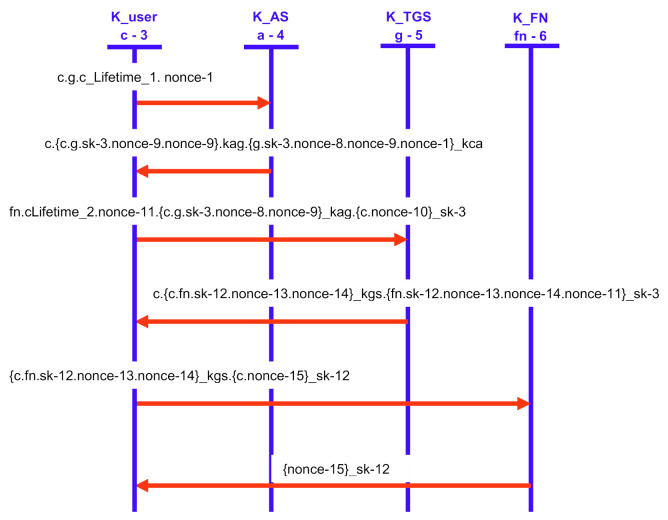
The simulation sequence using AVISPA.

Figure 11 and Figure 12 show our proposed scheme’s results with simulation results in OFMC and CL-AtSe back ends. These back-ends show that our scheme secure from active and passive attacks. The sequence diagram of the proposed scheme shown in Figure 13 is represented as user U, authentication server AS, ticket-granting server TGS, and fog node FN.

## 8. Security Features Comparison

The security features comparison is shown in Table 4. The schemes SAKA-FC [26] and AKA-FC [31] highly suffer from a key escrow problem and key encryption management. The scheme is also vulnerable to identity and password guessing attacks, replay Attacks, impersonation Attacks, insider Attacks, and DoS. Moreover, the cryptoanalysis shows that the scheme SAKA-FC [26] suffers from user anonymity and untraceability. The schemes SAKE [20] and AKA-FC [31] are vulnerable to stolen smart card attacks, offline password guessing attacks, and missing mutual authentications. The schemes SAKA-FC [26] and SAKE [20] do not facilitate perfect forward secrecy and biometric protection. Since the proposed scheme is validated using BAN logic, this ensures secure preservation of mutual-authentication and key session agreement. The proposed scheme is also simulated by the web tool AVISPA [40], whose simulation results indicate that it is defended against active and passive attacks. The proposed solution is protected against various security threats.

### 8.1. Computation and Communication Costs

Here, we explain the comparison of the communication cost and the computation cost of the proposed scheme with other existing schemes [20,26,31,32] which are shown in Table 5. The performance metrics can be explained as follows.

#### 8.1.1. Computation Cost

**Figure 14:**
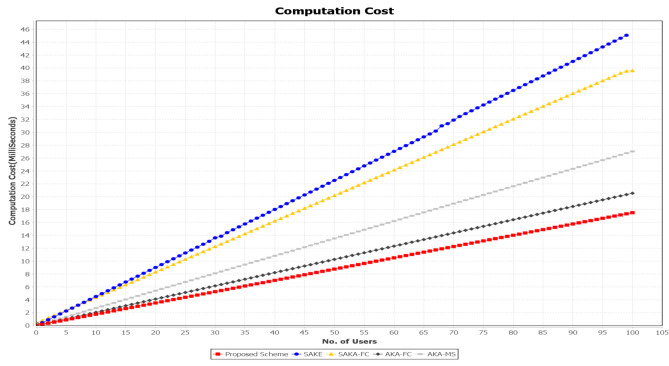
Computation costs comparison of SELAMAT against SAKA-FC, SAKE, AKA-FC, and AKA-MS.

In this subsection, we analyze the computation cost of the related authentication schemes SAKA-FC [26], SAKE [20], AKA-FC [31], and AKA-MS [32] and our proposed scheme. The number of cryptographic operations involved in this study are counted. To represent the comparison, Table 6 shows the notations, description, and computed their approximate execution time for various cryptographic operations by using the PBC library reported by Jia et al. [41]. Specifically, the study employed a secure hash function, public-key-based encryption, symmetrical encryption, and symmetric decryption, which are, respectively, denoted as $T_{H},\;T_{PE},\;T_{SE}$, and $T_{SD}$. It is noted that the XOR operation and concatenates operation ‖ are ignored because their execution time is negligible. The proposed scheme’s simulation was carried out on Intel Core™i7-5700HQ, CPU 2.70GHz platform using Java Pairing-Based Cryptography Library (JPBC) library. Figure 14 compares the proposed scheme’s computation cost against SAKA-FC, SAKE, and AKA-FC. Our scheme’s computation cost is shown in Table 5 comparing it to other authentication schemes.

In SAKA-FC [26], three cryptographic operation are scheme, $T_{sm}$, $T_{h}$, and $T_{mtp}$, respectively, as shown in Table 5. The execution times of these operations are 0.442, 1.709, and 4.406 ms, respectively. In the login phase, the user firstly needs to execute the Scalar multiplication $\left(T_{sm}\right)$ six times, Map-to-point hash function $\left(T_{mtp}\right)$ one, and the hash operation $\left(T_{h}\right)$ twenty-six times related to SAKA-FC to start login into the system, so the execution time of the phase costing nearly ≈35.595 ms. In the authentication phase, the user needs to execute the hash operations $\left(T_{h}\right)$ nine times related to $G_{1}$. Therefore, the execution time of the authentication process in SAKA-FC is ≈4.005 ms. Therefore, the total computation cost of their scheme is 39.595 ms. The computation of their scheme is computationally high due to the used multiplication operation in the scheme. In AKA-FC [31], there are three cryptographic operations related to ECC used in their scheme, $ET_{sm}$, $T_{h}$, and $T_{P}$, respectively. Table 5 shows the estimated execution time of the performed operations individually. However, in the login phase, the user needs to perform the scale multiplication related Elastic compute service (ECS) $2ET_{sm}$ twice, the hash function $4T_{h}$ four times, and the bilinear pairing $T_{P}$ once. Therefore, the execution time in the login phase is $2ET_{sm}+4T_{h}+T_{P}=9.251$ ms. In the authentication phase, the scheme needs to perform the scale multiplication related Elastic compute service (ECS) $2ET_{sm}$ three times, the hash function $4T_{h}$ eleven times, and the bilinear pairing $T_{P}$ once; thus, the time cost for this phase is $3ET_{sm}+11T_{h}+T_{P}=11.284$ ms. Therefore, the total computational cost of AKA-FC is 20.535 ms.

In SAKE [20], the scheme employed secure hash functions, multiplication, and fuzzy extractor operation are, respectively, denoted as $T_{h}$, $T_{pm}$, and $T_{fe}$ and are mainly related to fuzzy extractor algorithm. However, in the login phase, the user needs to execute point multiplication $T_{pm}$ twice, the hash function $8T_{h}$ eight times, and the fuzzy extractor operations $2T_{fe}$ twice. Consequently, the execution time in this phase is approximately 40.76 ms. In contrast, the authentication phase’s execution time is ≈4.295 ms, since the utilized operations are $T_{H}$ and $T_{pm}$. It is noted that the login phase takes longer than the authentication phase in this scheme under the usage of the multiplication operation. Therefore, the total execution time in the SAKE scheme is 45.055 ms. The AKA-MS scheme [32] uses two cryptographic operations: hash operation related to bilinear pairing $H_{P}$ and hash operation related to the group of ECC $H_{M}$. The estimated execution times are 12.418 and 0.974 ms, individually. The user needs to execute the hash operations $8H_{P}$ eight times in the login phase. In comparison, there is a need to execute the hash operations $7H_{P}$ seven times and the hash operation $1H_{M}$ once related to the ECC group in the authentication phase. The total computation cost is $8H_{M}+7H_{M}+1H_{p}=27.028$ ms, while the proposed scheme applied a very lightweight operation $T_{H}$, $H_{M}$, $T_{PE}$, $H_{P}$, $T_{SE}$, and $T_{SD}$. These operations’ execution times independently are 12.4180, 0.9740, 0.9740, 3.8500, 0.0046, and 0.0046 ms. However, in the first phase, the user needs to perform the hash function five times, public key encryption one time, the symmetrical encryptions four times, and the symmetrical decryption described as $1H_{P}+1H_{M}+5T_{H}+1T_{PE}+4T_{SE}$ twice. Therefore, the execution time of the login phase is nearly ≈17.2920 ms. In the authentication phase, the employed operations are $T_{H}$, $T_{AV}$, $T_{SE}$, and $T_{SD}$. The verification operation is included in this phase. However, the computation cost here is $2T_{H}+T_{AV}+2T_{SE}+3T_{SD}$. Therefore, the execution time for user operation is ≈0.232 ms. Thus, the total execution time of the proposed scheme is 17.524 ms. Compared to SAKA-FC [26], SAKE [20], AKA-FC [31], and AKA-MS [32], the proposed scheme has less computation cost. According to utilizing the AES-ECC algorithm, this result was achieved due to the fast AES encryption speed that makes it suitable for encryption of long plain-text. The ECC solution also uses a smaller key size and low computational system requirements, making it faster and more efficient cryptographic keys.

#### 8.1.2. Communication Cost

**Figure 15:**
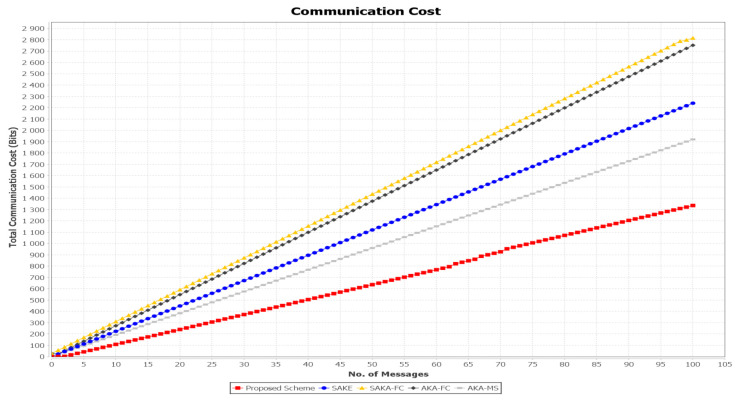
Communication costs comparison of SELAMAT against SAKA-FC, SAKE, AKA-FC, and AKA-MS.

The number of message interactions measures the communication costs. To compute the communication cost, it mainly depends on how many messages are transmitted between the entities multiplied by the (bit) size. We assume that the user’s identity can be represented by 32 bits, the secret value represented using 160 bits, the timestamp value is 24 bits, and the ticket value is represented as 128 bits. The communication cost of SAKA-FC [26], SAKE [20], AKA-FC [31], AKA-MS [32], and the proposed scheme are summarized in Table 5. The compassion of the proposed protocol’s communication cost against the selected works is shown in Figure 15. In SAKA-FC [26], the scheme exchanging messages $Msg.1=<RID_{i}^{{}^{\prime }},R_{u},a_{u},E_{u},F_{u},TS_{u}>$, needs (160+320+160+160+160+32)=992 bits, while in the login and authentication phase exchange the message $Msg.2=<RID_{i}^{*},RID_{k}^{{}^{\prime }},G_{i},H_{j},P_{f},TS_{f}>$ needs (160+160+160+160+320+32)=992 bits. The message $Msg.3=<RID_{k}^{*},M_{k},N_{k},P_{f},TS_{k}>$ needs (160+160+160+320+32)=832 bits. Therefore, the total communication cost of their scheme is 2×(992+992+832)=2816 bits. To evaluate the communication cost, the scheme AKA-FC [31] includes the length of the points in the group $G_{1}$, which is 1024 bits, the output of the hash function 2|q|, which has the length of 160 bits, and the length of the timestamp, being 32 bits, which is denoted as |T|. Thus, the communication cost in the login phase is 1376 bits. In the authentication phase, the user performs the same length of messages, which is represented as |G1|+2|q|+|T| and has the length of 1346. Therefore, the total communication cost of AKA-FC is 2752 bits. In SAKE [20], the initialization calculations on the user parameters set $(TS_{2}^{i}$, $ID_{RSU}^{i},O_{i},R_{i},Cert_{RSU}^{i})$. At this point, the total size of this message is calculated as 32 × 6 + 256 × 1 + 160 × 3 + 24 × 1 = 952 + 56 = 1008 bits. In the authentication phase, the server finally generates packet $\left(TS_{4}^{i},ID_{1}^{j},Cert_{RSU},\phi_{j}\right)$, Hence, the total communication cost for an individual user is (32 × 13) + (256 × 3) + (160 × 2 ) + (24 × 3) = 1576 bits. Therefore, the total communication cost for SAKE is 2584 bits.

In the AKA-MS scheme [32], the user exchanges the information (IDu,M,TW) with the registration center; hence, the total size is computed as (160 × 4)+ (256 × 1) + (32 × 1) = 928 bits. In the authentication phase, the user communicates with the server and finally exchanges $M_{1}=PID_{u},DID_{u}$, which needs (160 + 160) = 320 bits. Then, the server sends message $M_{2}=Q_{uj},V_{j}$, which has the size of (256 + 256) = 512 bits. The user later sends $M_{3}=Z_{uj}$ that needs 160 bits. Thus, the total communications cost of AKA-MS is 1920 bits.

In the proposed scheme, there are two interacting messages between the user and the authentication server in the login phase. The user first sends an initialization authentication request $Msg1.Auth_{u}^{{}^{\prime }}=E_{pk}\{u_{id}^{{}^{\prime }}\|X_{i}^{{}^{\prime }}\|Bio_{i}^{{}^{\prime }}\|tgs_{id}\left\|TS_{1}\right\}$; the size of the message is calculated as 32+160+32+32+24=248 bits. The AS sends the second message to the user as $Msg.2:(u_{id}^{{}^{\prime }}\|tgs_{id}\|E_{u_{pk}}\{ks_{\left(u\to tgs\right)}\|TS_{2}\left\|tgs_{tkt}\right\}$; the size of the message is computed as 32+32+128+24+160=376 bits. Therefore, the total communication cost in the first phase is (248+376)=624 bits. In the authentication phase, there are three messages which are shared between the user and the AS. The user sends a message to the TGS $Msg.3:Auth_{u\to tgs}=E_{ks_{u\to tgs}}\{u_{id}^{{}^{\prime }}\|fn_{id}\|TS_{3}\left\|tgs_{tkt}\right\}$, with the size of length computed as (32+32+24+160)=248 bits. Then, the TGS response to the user is calculated as $Msg.4:u_{id}\left\|fn_{tkt}\right\|E_{ks_{u\to tgs}}\left\{tgs_{id}\left\|ks_{{u\to fn}_{s}}\right\|ts_{4}\right\}$, with size (32+160+32+128+24)=376 bits. The user requests access to the fog node $Msg.5:Auth_{{u\to fn}_{s}}=E_{ks_{u\to fn}}\{u_{id}\|TS_{4}\left\|fn_{id}\right\}$; the message size is calculated as (32+24+32)=88 bits. Therefore, the total communication cost of the proposed scheme is (624 + 248 + 376 + 88) = 1336 bits. In our proposed scheme, the used operations are lightweight compared to the other schemes. Table 5 shows that the proposed scheme has a less communication cost compared to the others.

## 9. Conclusions

This paper proposes a lightweight multi-factor authentication scheme for cross-platform industrial IoT systems, *SELAMAT*. In SELAMAT, we use the AES-ECC algorithm for efficient and secure key management encryption mechanisms in the cloud provider server that acts as a trusted authority. Furthermore, the scheme adopts the Kerberos workflow due to the wide acceptance of the protocol in real-life applications. The designed algorithm offers secure communication between the edge devices and the fog node servers when the messages are transmitted via a public network. The proposed scheme enables edge devices to access any fog server in the fog computing network and improves the efficiency of the system by reducing the computation and communication cost to avoid a network burden.

The results show that the SELAMAT scheme reduces the communication and computation cost compared to the SAKA-FC, SAKE, AKA-FC, and AKA-MS schemes. This extensive comparison of the scheme efficiency shows that the proposed scheme can achieve better performance than the existing scheme. The AVISPA tool is used to verify the security of the scheme. The SELAMAT scheme provides robust security against attacks (replay attack, impersonation attack, man-in-the-middle attack, known-key attack, insider attack, server spoofing attack, etc.), and it was evaluated by using the formal and informal security analyses. In addition, the mutual authentication of the proposed scheme was proven by using the BAN logic. As mentioned above, the advantages pave a path for IIoT usability and suit the IIoT resources-constrained devices. In the future, the proposed scheme can improve the performance and the security of industrial hardware.

## Figures and Tables

**Table 1:** Comparison of the existing authentication scheme in fog computing.

Ref.	Issue	Structure	Implementation	Method	Performance	Limitation
[20]	Vulnerability to an ephemeral secret leakage attack.	Centralized	Prototype (IPhone6S (A9+M9highest 2GBCPU,2GB RAMandiOS10.11+PC).	ECC	Computation time = 85.7388 ms	Costly bilinear pairing operations, and Encryption key management problem.
[21]	Security issues in biometric mis-behavioral	Centralized	Prototype (ACOS and AET60 BioCARDKey kit)	AES	NA	Privacy issues may be used to track the individual and monitor the activities of the user.
[23]	Not preserving user privacy, therefore, exposed to MiTM attack	Centralized	PBC lab	PKC	Computational time = 387.762 ms	Public key revocation and Used a long-term master secret.
[26]	Fog user’s smart devices are resource-limited and cannot perform extensive, conventional digital signatures	Centralized	JPBC library and Bouncy Castle	Hash functions and symmetric encryption	Computation cost = 8.745 ms.	Vulnerability denial-of-service attack, replay attack, and password guessing attacks.
[26]	Several protection and privacy issues, including data exposure, session key leakage, replay, MiTM, and impersonation attacks	Centralized	NS2-simulation	ECC	Throughput, End-to-End Delay, Packet Loss Rate, Computation Costs= 54.124 ms	Not suite user authentication in a cloud driven IoT environment.
[12]	The application scenario is single and cannot be expanded to authentication between FC devices	Centralized	Java socket/MYSQL 5	AES	Communication cost = 5760 bits, Computation cost = 76.812 ms	GKM not efficient for mobile devices and require time-consuming computations.
[28]	Not suitable for mobile device deployment due to the requirement for computationally expensive operations	Centralized	JPBC library	HIDS	Computation Overhead = 30.871 ms, Communication Overhead = 2240 bits	The scheme needs more Needs more computational effort due to the identity-based cryptographic technique, Key escrow problem.
[33]	Heavy bandwidth consumption and high latency	Centralized	pbc-0.5.12	Homomorphic encryption	Total Computation time = 9.593ms, Communication cost = 2584 bits	Vulnerable to server spoofing attack and DoS attack.
[34]	limited resources of the edge node devices are requiring less computational resources	Centralized	Prototype (Raspberry Pi computer, and Matlab)	AES, and ECC	Power consumption, Data loss	Vulnerable to replay attacks.
[31]	Unprotected fog nodes in remote cloud data center	Centralized	MIRACL library	ECC	Total Computation time = 20.535 ms, Communication cost = 2752 bits	Vulnerable to man-in-the-middle attack, replay attack
[32]	Vulnerability to some attacks in the authentication between multi-servers	Centralized	Py-crypto library	ECC	Total Computation time = 27.028 ms, Communication cost = 1920 bits	Vulnerable to server spoofing attack, and lack of perfect forward secrecy

**Table 2:** Notation and abbreviations.

Notations	Description
SC	Smart Card.
$U_{i}$	User.
$u_{id}$	Identity of the User $U_{i}$
$u_{pw}$	The password of the user $U_{i}$.
$u_{bi}$	Biometrics imprint of the user $U_{i}$.
p,q	Two prime numbers.
$r_{i}$	Random number.
H(.)	One-way hash function.
E{.}/D{.}	A pair of symmetric encryption/decryption.
$Z_{q}^{*}$	The non-zero integers modulus p.
PK	The public key of the server.
*S*	The secret key of the server.
$Auth_{u}^{{}^{\prime }}$	The authenticator of the user $U_{i}$.
TS	Timestamp.
$ks_{u\to tgs}$	A key session between User and TGS.
$tgs_{sk}$	Secret key of the TGS.
$tgs_{id}$	Ticket granting server identity.
$fn_{id}$	Identity of the Fog node.
$tgs_{tkt}$	Ticket granting server ticket.
$ks_{u\to {fn}_{S}}$	Key session between User and FN.
$fn_{tkt}$	Fog Node ticket.
‖	Concatenation operation.
⊕	XOR operation.

**Table 3:** Notation and abbreviations.

Construct	Explanation
$U_{i}$	User.
AS	Authentication Server.
TGS	Ticket Granting Server.
FN	Fog Node.
$K_{X}$	X’s shared session key with CA.
$K_{X,Y}$	X’s shared session key with Y.
.K	A message encrypted by Key K.
$Ticket_{X,Y}$	Ticket used to visit X and Y.
$TS_{1},TS_{2},TS_{3}$	Random numbers Timestamp.
$X\overset{K}{\to }Y$	K is the shared session key between X and Y.

**Table 4:** Comparison on security properties.

	SAKA-FC [26]	SAKE [20]	AKA-FC [31]	AKA-MS [32]	SELAMAT
Key escrow	×	🗸	×	×	🗸
Replay attack	×	🗸	🗸	×	🗸
Impersonation attack	×	🗸	×	🗸	🗸
Man-in-the-middle attack	🗸	🗸	🗸	×	🗸
Known-key attack	🗸	🗸	🗸	🗸	🗸
Insider attack	×	🗸	×	🗸	🗸
Stolen smart card attack	🗸	×	🗸	🗸	🗸
Server spoofing attack	🗸	🗸	×	×	🗸
Denial of service (dos) attack	×	🗸	×	×	🗸
Offline password guessing attack	🗸	×	×	🗸	🗸
User anonymity	×	🗸	🗸	🗸	🗸
User untraceability	×	🗸	🗸	🗸	🗸
Mutual authentication	🗸	×	×	🗸	🗸
Perfect forward secrecy	×	×	🗸	×	🗸
Biometric protection	×	×	×	×	🗸
Cross-platform authentication	×	×	×	×	🗸

**Table 5:** Performance comparisons.

Scheme		Computational Cost	Communication Cost (bits)	Total
**SAKA-FC [26]**	Login phase	$2T_{sm}+26T_{h}+1T_{mtp}\approx 35.595$ ms	2240 bits	2816
	Authentication phase	$9T_{h}\approx 4.005$ ms	376 bits
**AKA-FC [31]**	Login phase	$2ET_{sm}+4T_{h}+T_{P}\approx 9.251$ ms	1376 bits	2752
	Authentication phase	$3ET_{sm}+11T_{h}+T_{P}\approx 11.284$ ms	1376 bits
**SAKE [20]**	Login phase	$8T_{h}+2T_{pm}+2T_{fe}\approx 40.76$ ms	1376 bits	2584
	Authentication phase	$20T_{h}+4T_{pm}\approx 4.295$ ms	1576 bits
**AKA-MS [32]**	Login phase	$8H_{M}\approx 7.792$ ms	928 bits	1920
	Authentication phase	$7H_{M}+1H_{p}\approx 19.236$ ms	992 bits
**SELAMAT**	Login phase	$1H_{P}+1H_{M}+5T_{H}+1T_{PE}+4T_{SE}+2T_{SD}\approx 17.292$ ms	624 bits	1336
	Authentication phase	$2T_{H}+T_{AV}+2T_{SE}+3T_{SD}\approx 0.232$ ms	712 bits

**Table 6:** Computation time consumption.

Description	Time (ms)
Identity-based signature $\left(T_{IDS}\right)$	23.866
Identity-based signature verification $\left(T_{IDV}\right)$	5.8720
Asymmetric signature $\left(T_{AS}\right)$	3.8500
Asymmetric signature verification $\left(T_{AV}\right)$	0.1925
Public-key-based encryption $\left(T_{PE}\right)$	3.8500
Public key-based decryption $\left(T_{PD}\right)$	3.8500
symmetrical encryption $\left(T_{SE}\right)$	0.0046
Symmetric decryption $\left(T_{SD}\right)$	0.0046
Scalar multiplication $\left(T_{\left(sm-ecc\right)}\right)$ in $G_{1}$	0.4420
Scalar multiplication $\left(T_{sm}\right)$	20.2300
ECS scalar multiplication $\left(ET_{sm}\right)$	1.9700
Exponentiation Operations $\left(T_{e}\right)$	1.2950
Bilinear pairing $\left(T_{P}\right)$	4.2110
Map-to-point hash function $\left(T_{mtp}\right)$	4.4060
Fuzzy extractor $\left(T_{fe}\right)$	0.0023
$H_{n}:{\left\{0,1\right\}}^{*}\to Z_{n}$	0.0023
$H_{P}:\left\{0,1\right\}\to G_{1}$	12.4180
$H_{M}:{\left\{0,1\right\}}^{*}\to G_{2}$	0.9740
$H_{S}:{\left\{0,1\right\}}^{*}\to {\left\{0,1\right\}}^{*}$	0.0046

## Data Availability

Data sharing not applicable.
